# Large-Scale Structure-Based Screening of Potential T Cell Cross-Reactivities Involving Peptide-Targets From BCG Vaccine and SARS-CoV-2

**DOI:** 10.3389/fimmu.2021.812176

**Published:** 2022-01-13

**Authors:** Renata Fioravanti Tarabini, Mauricio Menegatti Rigo, André Faustino Fonseca, Felipe Rubin, Rafael Bellé, Lydia E Kavraki, Tiago Coelho Ferreto, Dinler Amaral Antunes, Ana Paula Duarte de Souza

**Affiliations:** ^1^ Laboratory of Clinical and Experimental Immunology, Infant Center, School of Health Science, Pontifical Catholic University of Rio Grande do Sul (PUCRS), Porto Alegre, Brazil; ^2^ Kavraki Lab, Department of Computer Science, Rice University, Houston, TX, United States; ^3^ Antunes Lab, Department of Biology and Biochemistry, University of Houston, Houston, TX, United States; ^4^ School of Technology - Pontifical Catholic University of Rio Grande do Sul (PUCRS), Porto Alegre, Brazil; ^5^ Laboratório de alto desempenho – Centro de Apoio ao desenvolvimento cientifico e tecnológico da (IDEIA), Pontifical Catholic University of Rio Grande do Sul (PUCRS), Porto Alegre, Brazil

**Keywords:** SARS-CoV-2, structural bioinformatics, BCG, vaccine, cross-reactivity, HLA, peptide

## Abstract

Although not being the first viral pandemic to affect humankind, we are now for the first time faced with a pandemic caused by a coronavirus. The Severe Acute Respiratory Syndrome Coronavirus 2 (SARS-CoV-2) has been responsible for the COVID-19 pandemic, which caused more than 4.5 million deaths worldwide. Despite unprecedented efforts, with vaccines being developed in a record time, SARS-CoV-2 continues to spread worldwide with new variants arising in different countries. Such persistent spread is in part enabled by public resistance to vaccination in some countries, and limited access to vaccines in other countries. The limited vaccination coverage, the continued risk for resistant variants, and the existence of natural reservoirs for coronaviruses, highlight the importance of developing additional therapeutic strategies against SARS-CoV-2 and other coronaviruses. At the beginning of the pandemic it was suggested that countries with Bacillus Calmette-Guérin (BCG) vaccination programs could be associated with a reduced number and/or severity of COVID-19 cases. Preliminary studies have provided evidence for this relationship and further investigation is being conducted in ongoing clinical trials. The protection against SARS-CoV-2 induced by BCG vaccination may be mediated by cross-reactive T cell lymphocytes, which recognize peptides displayed by class I Human Leukocyte Antigens (HLA-I) on the surface of infected cells. In order to identify potential targets of T cell cross-reactivity, we implemented an *in silico* strategy combining sequence-based and structure-based methods to screen over 13,5 million possible cross-reactive peptide pairs from BCG and SARS-CoV-2. Our study produced (i) a list of immunogenic BCG-derived peptides that may prime T cell cross-reactivity against SARS-CoV-2, (ii) a large dataset of modeled peptide-HLA structures for the screened targets, and (iii) new computational methods for structure-based screenings that can be used by others in future studies. Our study expands the list of BCG peptides potentially involved in T cell cross-reactivity with SARS-CoV-2-derived peptides, and identifies multiple high-density “neighborhoods” of cross-reactive peptides which could be driving heterologous immunity induced by BCG vaccination, therefore providing insights for future vaccine development efforts.

## Introduction

Since March 2020, the coronavirus disease 2019 (COVID-19) pandemic caused by the Severe Acute Respiratory Syndrome Coronavirus 2 (SARS-CoV-2) has resulted in more than 5 million deaths globally (1). The virus is highly transmissible and infects mainly cells that express ACE2 receptors (2). The symptoms of COVID-19 vary widely, but typically include fever, dry cough, fatigue, and dyspnea (3). The eldelry and individuals with co-morbidities are at higher risk of severe disease, respiratory failure, and death. Several vaccines have been already approved, including vaccines based on messenger RNA, attenuated adenovirus, and inactivated virus (4). Despite the success of COVID-19 vaccines, mistrust and misinformation from segments of society, lack of resources in low-income countries, and impaired international coordination are all contributing to very limited vaccination coverage worldwide, a picture that will not change in the short-term. In addition, there is an increasing concern with new SARS-CoV-2 variants and the long-term effectiveness of the currently approved vaccines (5, 6).

The Bacillus Calmette-Guérin (BCG) is an old, live attenuated strain of Mycobacterium bovis. The BCG was originally formulated as a vaccine by Albert Calmette and Camille Guérin to prevent tuberculosis (7). This vaccine is safe, recommended for newborn children, and widely used in many countries (8). Interestingly, BCG vaccination has been reported to promote non-specific protection against other bacteria and viruses (9–12). Different mechanisms have been associated with this protection, also known as heterologous immunity (13), including epigenetics and trained immunity (14–16). Studies of heterologous immunity, as well as epidemiological data from vaccination studies, suggest that both infections and vaccines play a role in educating the immune system and that an optimal vaccination strategy can be beneficial to immune system maturation (17). In this context, several studies considered whether the BCG vaccine could provide partial protection against SARS-CoV-2 infection. Currently, there are 28 registered clinical trials testing the efficacy of BCG in attenuating COVID-19 (18). Several epidemiology and ecologic studies have been conducted to associate the vaccination of BCG with protection to COVID-19. However the data are controversial (11, 19–29). Studies comparing the mortality rates between different countries presented a drawback related to the inaccuracy of the reported mortality data in some countries. One epidemiologic study pointed out that BCG may offer some protection against COVID-19 (30). Still, the data should be interpreted with caution as it may depend on the time of pandemic and the age structure of the population vaccinated with BCG. A Phase III randomized clinical trial confirmed that recent vaccination with BCG protects the elderly against new respiratory infections (31). Also, booster BCG vaccination in high-risk healthcare workers was shown to prevent COVID-19 (32). Accordingly, in the mice model, BCG administration can protect from SARS-CoV-2 infection (33).

It is known that the individual repertoire of induced T cells (i.e., private specificity), shaped by previous infections and vaccinations, determines immunopathology and heterologous immunity (34). Therefore, the protection of BCG against COVID-19 might be mediated by cross-reactive T cells. T cells recognize peptides displayed by class I Human Leukocyte Antigen (HLA-I) receptors, and a recent study analyzing HLA-I-restricted peptides has shown sequence similarity between BCG and SARS-CoV-2 epitopes (35). Also, the study of Eggenhuizen et al., identified 8 BCG vaccine-derived peptides with sequence homology to SARS-CoV-2 NSP3-derived peptides (36). In a study with 20 individuals, they found that cells primed with BCG peptides developed enhanced T cell reactivity to 7 SARS-CoV-2 peptides (36). T cell cross-reactivity is not an uncommon event and was already described in the context of coronavirus infection (37). The hypothesis that cross-reactive T cells primed with BCG may be involved in the response against SARS-CoV-2 has already been discussed, and supported by *in silico* studies focused on peptide sequence similarity (38). However, T cell cross-reactivity is not determined only by sequence similarity. Recent studies have demonstrated that T cell cross-reactivity is also determined by structural similarities of unrelated peptide-HLA (pHLA) complexes (39–41).

In the present study we aimed at performing a structure-based screening for potential cross-reactive HLA-I-restricted peptides from BCG and SARS-CoV-2. We performed a large-scale proteome analysis and identified thousands of possible HLA-I binders. We modeled these peptides in the context of different HLAs, and used a large-scale image-based analysis to compute similarity between pHLA complexes, while accounting for biochemical and structural properties. This analysis produced a short list of immunogenic BCG-derived peptides that are the most likely primers for T cell cross-reactivity against SARS-CoV-2. It also produced a longer list of cross-reactivity clusters involving one SARS-CoV-2 peptide and multiple BCG-targets, in some cases binding multiple HLA-I alleles, which could represent interesting targets for vaccine development.

## Material And Methods

### Peptide and HLA Selection

We obtained the proteomes from BCG-Pasteur strain (ID: UP000032723, hereafter called BCG) and SARS-CoV-2 (ID: UP000464024, hereafter also called SARS) from the UniProtKB database. We applied a sliding window method on each protein aiming to generate all possible peptides with 9 amino acids in length (redundant peptides were removed). The HLAs were selected according to prevalence in the human population and information on literature about SARS-CoV-2 related HLAs (http://pypop.org/popdata/) (42). We end up with a total of 10 HLAs: HLA-A*01:01, HLA-A*02:01, HLA-A*02:02, HLA-A*11:01, HLA-A*24:02, HLA-B*07:02, HLA-B*15:03, HLA-B*35:01, HLA-B*40:01, and HLA-B*51:01.

### Filtering by HLA Binding

We filtered peptides according to HLA-I binding affinity. This step defined the set of pHLA complexes that should be modeled in the next phase. The implementation of this phase uses the HLA-Arena tool (43). After setting up our environment, we implemented different processing pipelines, each one on a separate python script, using as reference the “virtual screening” notebook from HLA-Arena (i.e., 3_virtual_screening.ipynb). We integrated individual python scripts into a single script (i.e., run.sh) to parallelize the jobs. This script uses as input (i) 10 HLA-I alleles, (ii) 9,814 peptides from SARS-CoV-2, and (iii) 1,242,895 peptides from BCG as fasta/regular text files. After that, we first generate a list of all possible pHLA complexes and run the MHCflurry (44), generating a list of pHLAs with the respective peptide binding affinity. Secondly, we filter only the set of complexes that present a binding affinity equal to or lower than 500 nM. We set this threshold aiming to recover only strong and intermediate HLA-I binders. There is also evidence that there is a correlation between peptides below this threshold and immunogenicity (45). This set of complexes is selected to be modeled in the next phase. The result is stored in 28 files for each combination between the list of alleles and BCG or SARS-CoV-2 peptides (e.g., HLA-A0101_BCGpeps, HLA-A0101_SARSpeps; HLA-B0702_BCGpeps, HLA-B0702_SARSpeps). All scripts and files were made freely available in GitHub and more information can be found in Supplementary Data.

### Structural Modeling

The structural modeling of the selected pHLAs also uses the HLA-I Arena tool and is based on the “virtual screening” notebook implemented in the tool. The phase is divided into three steps. The first step uses the MODELLER tool (46) to create an HLA-I structure for each of the 10 HLA-I alleles selected. The second step models the pHLA using the APE-Gen tool (47). The third step involves the management of big data, because we needed to parallelize the whole process in a limited number of machines. To manage the execution of all batches and handle file transfers between machines, we developed a scheduler in Python called ArenaDispatcher (see Supplementary Data). The ArenaDispatcher uses the Docker API to communicate with docker daemons on either local or remote machines and can schedule jobs in the form of containers to run on one of the connected docker daemons. The ArenaDispatcher also monitors the execution state of each job, schedules in accordance to a maximum (or unlimited) amount of available resources for each docker host, and retrieves data (files and directories) and execution logs (i.e., stdout and stderr). We also included problem circumvention functionalities such as rescheduling jobs of unavailable hosts, reconnecting to previously unavailable hosts, and a local log file for the dispatcher’s consistency (in case of a crash, it can reload the information of jobs completed, running, or not yet dispatched). By doing this, associated with cloud resources (i.e., Amazon Web Services acquired utilizing a grant from Brazilian National Research and Education Network), we reduced the time of simulation from 7 years to approximately 60 days.

### Generation and Extraction of Surface Data From pHLA Complexes

The PyMOL tool (https://pymol.org) was used to generate a visual representation of the TCR-interacting surface electrostatic potential of each pHLA complex. A script was developed to automate this process for each PDB file, exporting the result in the format of PNG images after the alignment of the complex from a pivot point, assuring all pHLA complexes would be in the same orientation (i.e., same x, y, z coordinates). The ImageJ tool (https://imagej.nih.gov/ij/) was used to obtain the histogram containing RGB values (i.e., mean and standard deviation) of 46 regions of interest (**Figure 2A**) in each of these images. Given the large number of images and the ImageJ limitation in obtaining the histogram of one region at a time, we developed a plugin in Java (see histogram2csv in Supplementary Material). The plugin receives an image and a Region of Interest (ROI) Set, and generates the histogram for each region of interest through command line. The result is exported in a CSV format file, where each line corresponds to a region of interest and each column corresponds to information from the histogram of that region.

### Hierarchical Clustering Analysis

To evaluate the potential of cross-reactivity between pHLA complexes, the electrostatic potential of the TCR-interacting surface was submitted to a hierarchical clustering analysis, similar to what has been done in (48). Briefly, after modeling the pHLA with PyMOL and extracting the RGB values of each complex, we used pvclust (version 2.2) (49) and fastcluster (version 1.1.25) packages (50) from R software (version 3.6.1) (https://www.r-project.org/) (51) to perform a hierarchical clustering analysis. To perform the clustering we used the pvclust parameters method.hclust = “single” and method.dist = “correlation”. The single linkage method was used to reduce memory usage and computational time. The procedure was replicated 1,000 (default nboot value for pvclust) times per HLA-I allele.

### Biochemical Properties Analysis

Peptide features can be grouped upon residue physical and chemical similarities. To sample the most similar SARS-CoV-2 and BCG peptides in our analysis, we filtered candidates based on amino acid size, hydrogen bond donors and acceptors, charge, aliphaticity, and aromaticity. In short, our strategy compares a weight vector related to biochemical properties for SARS (query) and BCG (subject) peptides. As a result, a peptide similarity ranking is produced spanning the whole dataset of SARS-CoV-2 and BCG pairs for each HLA-I allele. Finally, the peptide ranking per allele was filtered and we considered 10,000 pairs for downstream analysis.

### Filtering Putative Cross-Reactive Clusters

Dendrograms are broad structures that report several clusters in distinct levels. The closest levels are related to clusters with higher similarity, i.e., in our case indicate putative cross-reactions. To filter closest cross-reactive clusters, we adopt a depth-first search (DFS) approach to explore the adjacent branches in each HLA-I allele data. Firstly, the dendrograms were converted to a graph-based structure. Next, we develop a DFS-based algorithm that identifies the closest clusters using the SARS-CoV-2 peptides as a seed node. The search depth was arbitrarily limited to five branches or bipartitions (d <= 5). The DFS algorithm was developed in R using functions derived from igraph version 1.2.6 (52) and ape packages version 5.5 (53).

### Cluster Prioritization and Experimental Evidence

To provide additional information, experimental data on SARS-CoV-2 peptides were integrated into cross-reactive clusters. The data was retrieved from IEDB (iedb.org) and we used filters to recover only HLA-I peptides that were recognized by T cells (i.e., immunogenic peptides) in humans. Peptide immunogenicity data were used to select the interaction pairs among all HLA-I alleles.

### Computational Resources and Data Availability

The HLA-I filtering and modeling processes were executed in a single machine composed of 24 cores and 192 GBytes of memory. Each core uses hyper-threading, which provides two virtual (logical) cores (or execution threads). Therefore, the cluster has in total 192 virtual cores for processing. Analyzing the resource utilization of APE-Gen and aiming for the best parallelization, we used containers configured with 6 virtual cores, resulting in 32 containers in the cluster and 100 complexes per execution. To accelerate the performance, we used virtual machines from Amazon Web Services acquired utilizing a grant from RNP (Brazilian National Research and Education Network) dedicated for COVID-19 research. Each virtual machine was configured with 8 cores and 15 GB of memory (c4.2xlarge instance). We used 8 virtual machines. In the end, using local and cloud resources, we concluded the modeling of all complexes in approximately 60 days. The final result was 358,386 pdb files representing the modeled complexes. Each pdb file has approximately 480Kbytes, giving a total of 160 GBytes. For the image analysis we generated 358,383 png files (three pHLAs caused PyMol to fail and crash) occupying 257 GBytes of storage space. After concluding the surface generation with PyMol, ImageJ plugin processed sequentially every png file in approximately 12 hours, storing the extracted information on CSV (comma-separated values) files (each one with 12 KBytes) requiring 4,3 GBytes of storage space. The complete list of modelled pHLAs, fasta files, png files, plugins, and computational scripts are available at https://github.com/LAD-PUCRS/Arena_SARS-BCG. The structural data is also available upon request.

## Results

**Figure 1:**
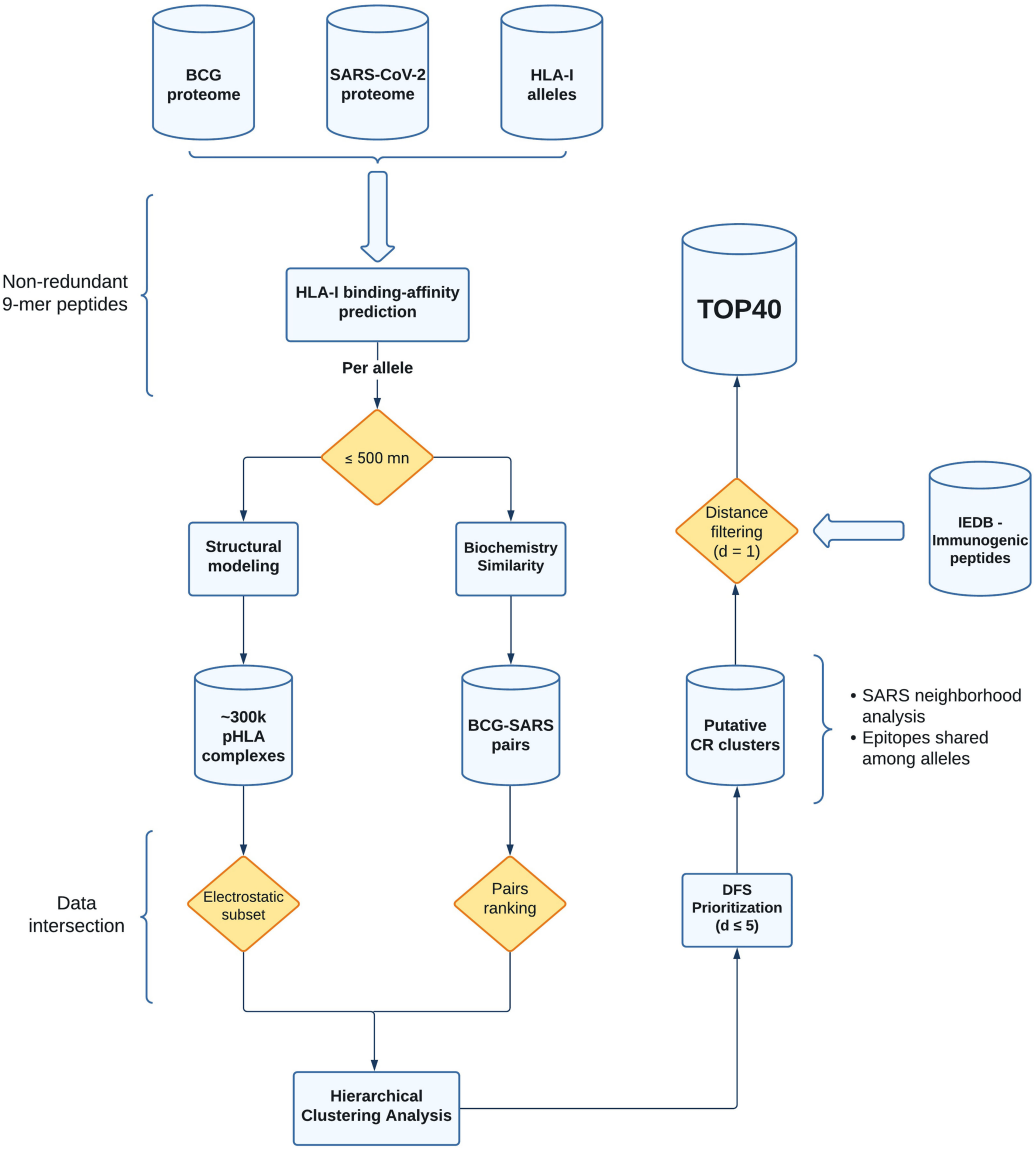
Flowchart with all the steps from data acquisition to processing and selection of the putative cross-reactive clusters. The shapes in the flowchart represent distinct aspects of our pipeline. Cylinders and rectangles are showing database (output storage) and computational analyses (including third-party tools, see *Material and Methods*), respectively. The yellow diamonds represent data filtering (pHLA binding affinity, electrostatic subset, pairs ranking, and distance filtering). The curly brackets are showing additional analysis or parameters chosen in our study. BCG, Bacillus Calmette-Guérin (Pasteur strain); SARS, SARS-CoV-2; HLA-I, Human Leukocyte Antigen of class I; pHLA, peptide-HLA; DFS, Depth-first search; CR, cross-reactive; IEDB, Immune Epitope Database.

We have implemented a new computational strategy to select putative cross-reactive peptides between BCG and SARS-CoV-2. Starting from the entire proteome of both viruses, our methodology allowed for the identification of 40 pairs of peptides with higher probability to trigger cross-reactive T cell responses. These sequences were selected through a series of filters considering affinity to multiple prevalent class I HLA-I alleles, and pHLA similarity in terms of biochemical and structural features. The entire workflow is summarized in **Figure 1**.

### Selection of Potential HLA-I Binders From BCG and SARS-CoV-2 Proteome

We hypothesize that partial heterologous immunity between BCG and SARS-CoV-2 can be mediated by T cell cross-reactivity between similar viral peptides displayed by HLA-I molecules. In order to identify candidate peptide-targets for these cross-reactive responses, we started by fetching the whole proteome of BCG and SARS-CoV-2 from UniprotKB. In total, we analyzed 3,891 proteins from BCG, and 14 proteins from SARS-CoV-2. We also searched the literature and public databases for HLAs with high population coverage, as well as those reported to be involved in SARS-CoV-2 immune responses. We restricted our analysis to a total of 10 HLAs (i.e., five HLA-A and five HLA-B alleles). Since HLA-I molecules most frequently present peptides of nine amino acids (9-mers) in length (54), we further recovered all possible 9-mers from BCG and SARS-CoV-2 proteome. This resulted in a total of 1,237,282 (BCG) and 9,814 (SARS-CoV-2) peptides.

Since not all 9-mers would be able to bind all HLA-I molecules, according to HLA-I binding restrictions, we further filtered these peptides using a sequence-based binding affinity predictor. For this step, we set a threshold of 500 nM aiming to recover only strong and intermediate HLA-I binders (45). As a result, we recovered a total of 296,651 and 2,918 peptides from BCG and SARS-CoV-2, respectively. Each peptide was modelled in the context of its specific HLA using APE-Gen (47) and HLA-Arena (43), generating a total of 358,386 pHLA structures. A small percentage of complexes could not be modeled by APE-Gen, and was removed from the analysis. The calculations were performed at PUCRS High Performance Computing Lab (LabLAD) and all the data is made freely available upon request. This represents one of the largest repositories for three-dimensional structures of pHLA complexes available.

### Selection of Similar Peptides Based on Biochemical Properties

To further refine our analysis to consider only the most similar pairs of peptides (i.e., BCG-SARS pairs) we apply a filter based on biochemical properties (BP). For that, we represented each peptide as a biochemical properties (BP) vector reflecting its amino acid sequence, as previously described in (55). The L1 norm was used to score the overall differences between each SARS-CoV-2 and BCG peptides displayed by the same HLA-I allele, respectively. Next, we rank BCG-SARS pairs based on the BP score. The top 10,000 BCG-SARS pairs across all lists of the same HLA-I allele were selected for further analysis. Note that the number of recovered peptides varied across HLA-I alleles. For instance, most of the peptides selected are presented by the HLA-B*15:03 (13.5%), while HLA-B*51:01 is the HLA-I allele with the lowest number of peptides (2.1%).

After removing redundant peptides, we obtained 55,536 BCG-derived peptides that were biochemically similar to 2,113 peptides derived from SARS-CoV-2. The per-allele totals can be found on **Supplementary Table 1**.

### Selection of Similar pHLAs Based on Structural Features

Although peptide similarity in terms of biochemical properties can suggest a higher probability for cross-reactivity (56–58), T cell cross-reactivity is also driven by structural features of the pHLA complex (e.g., topography and electrostatic potential over the TCR-interacting surface) (39). To account for those additional features, we included all the complexes selected in the previous step (i.e., a total of 57,649 pHLA complexes, divided into 10 HLA-I alleles) into an HLA-restricted pairwise structural comparison analysis. Briefly, we extracted the image of the TCR-interacting surface of each modeled pHLA complex, capturing its topography and electrostatic potential.

**Figure 2:**
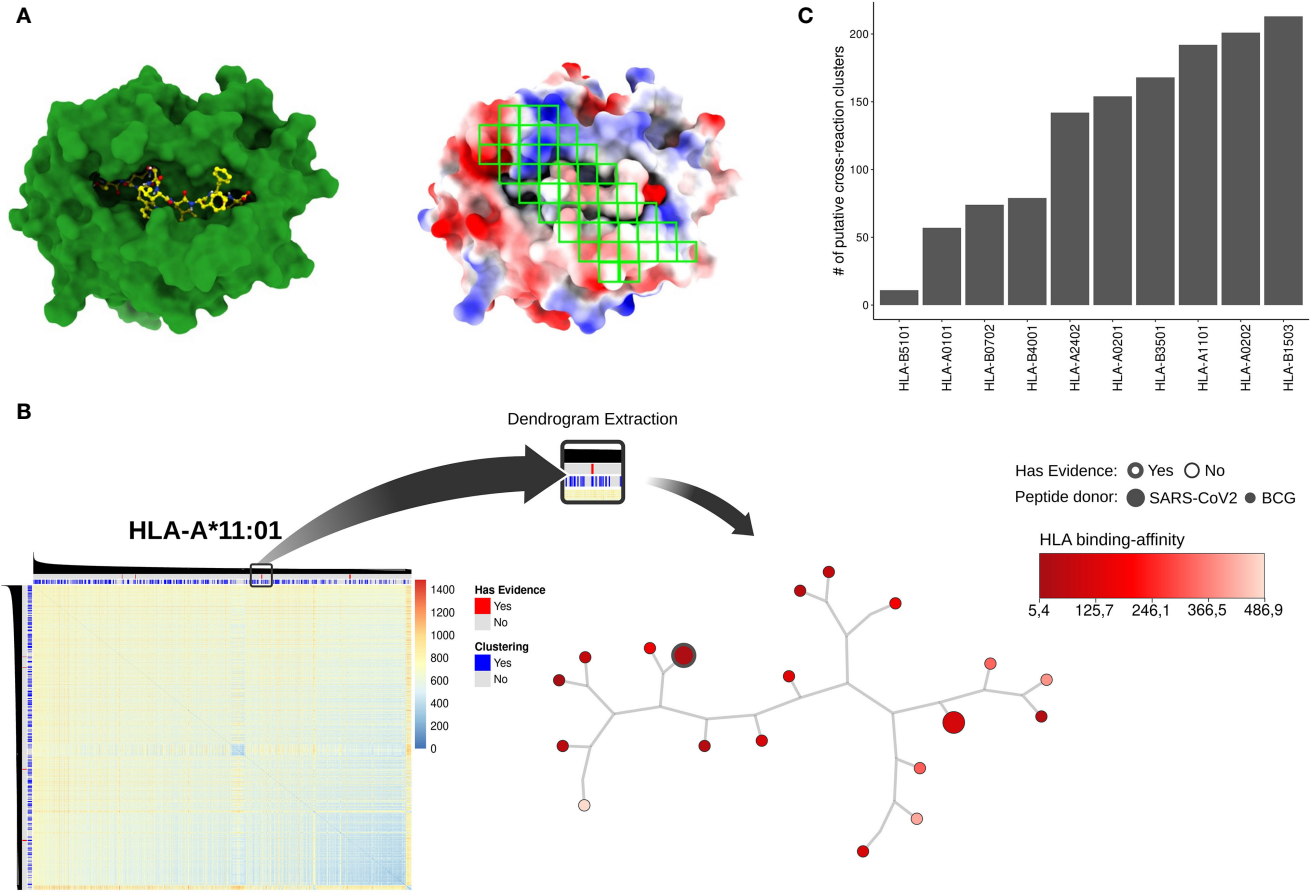
**(A)** pHLA models using HLA-A*02:01 and the SARS-derived peptide TQWSLFFFL as an example. On the left, the HLA-I alpha chain and the peptide are represented in green and yellow, respectively; on the right, forty-six regions (green squares) were sampled for electrostatic potential analysis through RGB mean and standard deviation values extraction. The red, white, and blue represent negative, neutral, and positive charges, respectively. The electrostatic potential range used was -3kT to +3kT. **(B)** An overview related to dendrogram extraction based on the DFS algorithm. As an example, the HLA-A*11:01 heatmap was obtained after the HCA procedure. The color key represents Euclidean distance applied as the similarity metric into dendrogram clustering. The color gradient blue to red indicates high to lower similarity among pHLA structures, respectively. On the superior bars the clustering (i.e., grouping pHLA by similarity using our approach) and experimental evidence status (i.e., data gathered from literature) are represented. SARS-CoV-2 peptides with experimental support are shown in red, and the DFS-based cross-reactive clusters are shown in blue. The tree-base structure in the right corner represents a filtered cluster in HLA-A*11:01 data. The predicted binding affinity (i.e., how well the peptide binds that specific HLA) is shown in the red color key. The node size represents the peptide-donor (i.e., SARS-CoV-2 or BCG) and the silhouette thickness indicates if the peptide is immunogenic, according to the IEDB database. In **(C)** we recover the total number of cross-reactivity clusters for each one of the HLAs present in our analysis.

Following a similar protocol to that described in previous studies (48), we determined 46 gates over this image (**Figure 2A**). These gates were designed to cover known “hot-spots” for T cell interaction derived from crystal structures (i.e., curated interactions from IEDB) and previous studies (48). We then extracted summarizing statistics from each of these gates to create a vector representing each complex. Finally, these vectors were used as input for a Hierarchical Clustering Analysis (HCA). This strategy produced 10 dendrograms (i.e., one for each HLA-I allele) summarizing all structure-based pairwise comparisons of pHLA complexes (**Figure 2B** and **Supplementary Figure 1**).

A popular way to visualize these pairwise comparisons is through the use of heatmaps, but they became harder to interpret for larger datasets. Here, we traverse the dendrogram using a DFS approach with a cut-off distance lower or equal to 5 (see *Material and Methods*). We arbitrarily choose this value aiming to recover clusters of possible cross-reactive targets between SARS-CoV-2 and BCG peptides. From this analysis, we retrieved a total of 16,606 putative BCG-SARS cross-reactions (**Supplementary Table 2**), distributed throughout a diverse number of clusters per-allele (**Figure 2C**). This database of putative cross-reactive clusters (**Figure 1**, see Putative CR clusters) was then used for two different analyses, presented in the following sessions. First, we conducted a qualitative analysis focused on available experimental data (i.e., identifying experimentally-determined immunogenic peptides from IEDB (59). Second, we performed a quantitative analysis driven by the data. Here we computed (i) the “density” of BCG pHLA-targets in close distance to SARS-CoV-2 pHLAs, and (ii) the overlap degree of SARS peptides within cross-reactive clusters displayed by distinct HLA-I alleles (**Figure 1**).

### Identification of Cross-Reactive Clusters Including Immunogenic Peptides From BCG Vaccine

Immunogenicity, defined as the capacity of a peptide to trigger an immune response, is one of the most important features in epitope discovery. It is also a requirement for potential involvement in T cell cross-reactivity. Therefore, we initially focused on identifying SARS-CoV-2 peptides present in our putative cross-reactivity clusters, defining three conditions: i) a DFS distance equal to one, plus experimental evidence regarding ii) HLA-I binding-affinity matching to our prediction, and iii) T cell response assay reported by on IEDB. After this filtering, we produced a table of 40 possible cross-reactive BCG-SARS pairs (**Table 1** and **Supplementary Table 3**). In summary, according to our approach, these immunogenic SARS-CoV-2 peptides are highly similar to BCG peptides, both in terms of biochemical properties as well as structural pHLA features (**Supplementary Figure 4**).

**Table 1:** Top 40 putative BCG-SARS cross-reactions.

HLA predicted	Peptide source		DFS distance	SARS-CoV-2 peptide presented by	Protein source
	SARS-CoV-2*	BCG			
HLA-A*01:01	CNDPFLGVY	MTAFGVEPY	1	HLA-A*0101	SPIKE
HLA-A*01:01	FSAVGNICY	WTDVKFALI	1	HLA-A*01:01	R1A, R1AB
HLA-A*01:01	GTDLEGNFY	EVDSAFDGY	1	HLA-A*01:01	R1A, R1AB
HLA-A*02:01	FIAGLIAIV	TLAGLLPPV	1	HLA-A*02:01	SPIKE
HLA-A*02:01	FVFLVLLPL	WVFLVNLPL	1	HLA-A*02:01	SPIKE
HLA-A*02:01	GLMWLSYFI	TMWLHVPAV	1	HLA-A*02:01	VME1
HLA-A*02:01	ILGLPTQTV	IVAALLVTI	1	HLA-A*02:01	R1AB
HLA-A*02:01	IVAGGIVAI	NLTGGIVAL	1	HLA-A*02:01	R1A, R1AB
HLA-A*02:01	KLVNKFLAL	KMAKSVLLA	1	HLA-A*02:01	R1A, R1AB
HLA-A*02:01	TLMNVLTLV	ALVLNLLPI	1	HLA-A*02:01	R1A, R1AB
HLA-A*02:01	YLASGGQPI	LLSAAGVPL	1	HLA-A*02:01	R1A, R1AB
HLA-A*11:01	ATVVIGTSK	GLNVNTLSY	1	HLA-A*11:01	R1AB
HLA-A*11:01	VVNARLRAK	IVASRGAQK	1	HLA-A*11:01	R1AB
HLA-A*24:02	AYANRNRFL	VWAQVRNRL	1	HLA-A*24:02	VME1
HLA-A*24:02	IFFITGNTL	TFGALAITL	1	HLA-A*24:02	R1A, R1AB
HLA-A*24:02	MFTPLVPFW	IYPPQVALV	1	HLA-A*24:02	R1A, R1AB
HLA-A*24:02	TFNGECPNF	EYLETIHTW	1	HLA-A*24:02	R1A, R1AB
HLA-A*24:02	VFVSNGTHW	SYIAYAPQL	1	HLA-A*24:02	SPIKE
HLA-A*24:02	VYMPASWVM	LYGIFIVWL	1	HLA-A*24:02	R1A, R1AB
HLA-A*24:02	YFMRFRRAF	AWRRLTKVI	1	HLA-A*24:02	R1A, R1AB
HLA-A*24:02	YFPLQSYGF	GWPTWGMIL	1	HLA-A*24:02	SPIKE
HLA-B*07:02	LPNNTASWF	APNSGLVAA	1	HLA-B*07:02	NCAP
HLA-B*07:02	QPGQTFSVL	FPMLQFSLL	1	HLA-B*07:02	R1A, R1AB
HLA-B*07:02	RARSVSPKL	RAATAAMVM	1	HLA-B*07:02	NS7A
HLA-B*07:02	TPRDLGACI	LVVDAARAM	1	HLA-B*07:02	R1A, R1AB
HLA-B*40:01	GEAANFCAL	TEVLAAQHL	1	HLA-B*40:01	R1A, R1AB
HLA-B*40:01	GEVITFDNL	SEVVVFDAA	1	HLA-B*40:01	R1A, R1AB
HLA-B*40:01	SELLTPLGI	AEMTVALLL	1	HLA-B*40:01	R1A, R1AB
HLA-B*40:01	WEPEFYEAM	AELEAQQEL	1	HLA-B*40:01	R1AB
HLA-A*11:01	QVVDMSMTY	AQPTEPVLK	1	HLA-A*01:01, HLA-A*11:01	R1A, R1AB
HLA-A*11:01	SASKIITLK	ASGAKTGAK	1	HLA-A*03:01, HLA-A*11:01	AP3A
HLA-A*24:02	VYFLQSINF	VYSVLLALL	1	HLA-A*24, HLA-A*24:02	AP3A
HLA-A*24:02	YFVVKRHTF	VFPGRKGGF	1	HLA-A*24:02, HLA-B*08:01	R1AB
HLA-B*07:02	FPRGQGVPI	DPRGNPVPL	1	HLA-B*07:02, HLA-B*08:01	NCAP
HLA-B*35:01	FAYANRNRF	MASAARLAA	1	HLA-B*15:01, HLA-B*35:01	VME1
HLA-B*35:01	SANNCTFEY	QPALFTVEY	1	HLA-A*29:02, HLA-B*35:01	SPIKE
HLA-B*40:01	NELSRVLGL	VEGQTNHML	1	HLA-B*40:01, HLA-B*44:02	R1A, R1AB
HLA-B*35:01	TSNQVAVLY	IAAMLLVIY	1	HLA-A*26:01, HLA-B*35:01, HLA-B*57:01	SPIKE
HLA-B*40:01	AEIRASANL	AEPRATGHI	1	HLA-B*40:01, HLA-B*44:02, HLA-B*44:03	SPIKE
HLA-A*01:01	LTDEMIAQY	MTNDNLEYY	1	HLA-A*01:01, HLA-A*29:02, HLA-B*35:01, HLA-C*07:02	SPIKE

*Immunogenic SARS-CoV-2 peptides.

### Cross-Reactivity Prediction Between BCG and SARS-CoV-2

Since there is currently no experimental support for the immunogenicity and cross-reactivity of most peptides in our predicted cross-reactivity clusters, we decided to also perform a quantitative analysis to explore other ways to prioritize potential targets of cross-reactivity in our data. In this context, we performed two different analyses: cluster density and HLA-I restriction.

In the first case, we focused on the “density” of BCG pHLA-targets in close distance to SARS-CoV-2 pHLAs (indicated in **Figure 1** as SARS neighborhood analysis). The density was calculated based on a number of BCG peptides within the cross-reactivity cluster (i.e., SARS-BCG pairs inside d <= 5). As a result, several high-density SARS neighborhoods were identified across HLA-I alleles. The SARS pHLAs with high-density neighborhoods are an interesting resource due to the higher potential of triggering heterologous immunity with several BCG peptides. Finally, these findings can be used for further investigation of potential cross-reactivity. **Supplementary Figures 2**, **3** show 50 SARS neighborhoods for each HLA-I allele in this study.

**Figure 3:**
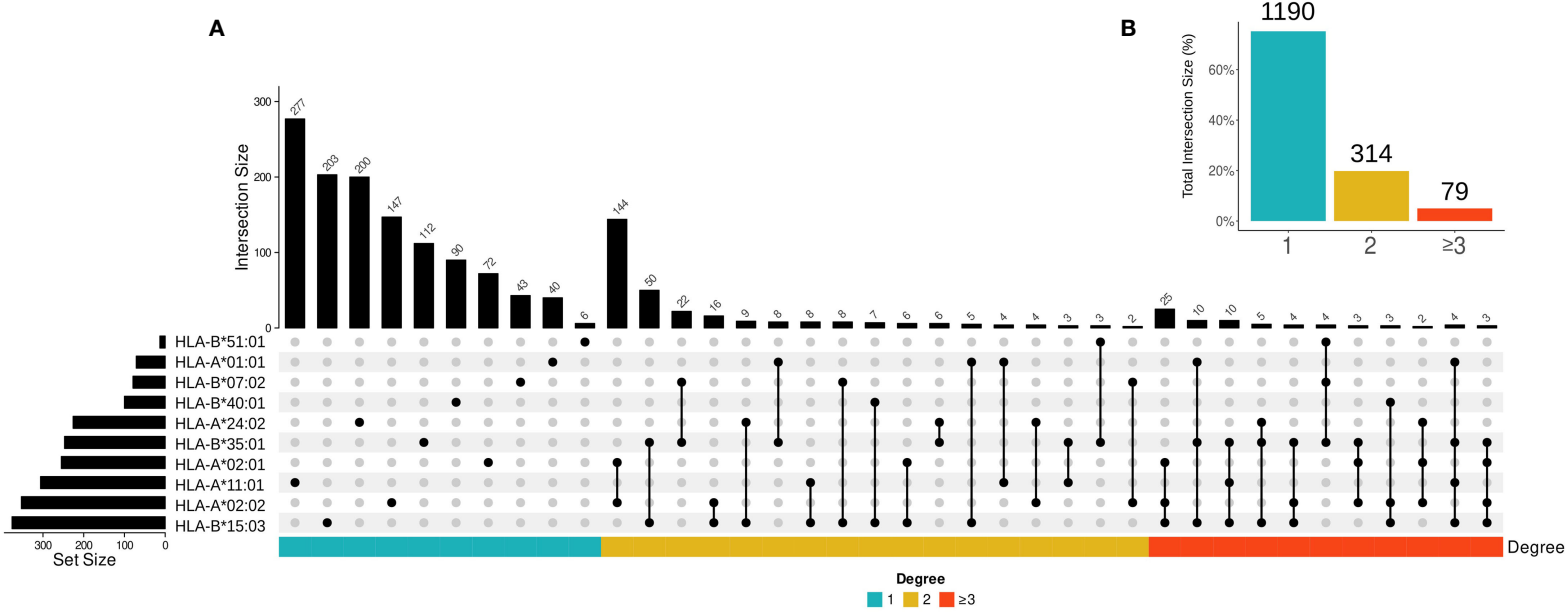
The overall profile of cross-reactive SARS-CoV-2 epitopes presented by different HLA-I alleles. **(A)** The intersection size represents the number of SARS-CoV-2 epitopes per cross-reactivity cluster when presented by one, two, and three or more HLAs. The edges connecting HLA-I alleles represent an intersection among SARS-CoV-2 epitopes (i.e., the number of epitopes presented by the same HLA molecule). Only the intersections higher or equal to two are shown. The set size represents the total number of SARS-CoV-2 epitopes in each HLA-I allele. **(B)** The percentage of shared SARS-CoV-2 epitopes per HLA-I allele bins is shown. The color bar represents intersection degree bins in each plot.

In the second case, we examined whether SARS-CoV-2 epitopes in putative cross-reactive clusters can be presented by multiple HLA-I alleles using UpSetR to create visualizations of intersecting sets (**Figure 3A**) (60). Epitopes presented by multiple HLA-I alleles are promising resources for vaccine design and immunotherapy, since they maximize population coverage (61, 62). As expected, our results show a higher percentage of putative cross-reactive epitopes presented by a single HLA-I allele, and a lower percentage of epitopes shared between 2 or more than 3 HLA-I alleles (**Figure 3B**).

Note that there is not a clear correlation between the number of peptides presented by one allele and the number of putative cross-reactive clusters. For instance, most of the peptides are presented by HLA-A*11:01, while the largest number of cross-reactive clusters was observed for epitopes restricted to HLA-B*15:03. In terms of shared cross-reactive clusters, both HLA-B*15:03 and HLA-B*35:01 are the most represented alleles, as observed in **Figure 3** by the number of connections to other HLA-I alleles (16 and 14 times, respectively).

## Discussion

The development of functional memory T cell response is a complex process, which plays a fundamental role in vaccine-induced protection. Pre-existing memory T cells can be activated by cross-reactive peptide-targets (63), making response to immunization to be largely driven by individual immunological history (64). For instance, a study with yellow fever virus showed that T cell kinetics after vaccination depended on the pre-vaccination precursor frequencies and initial differentiation states (65). Their results indicate that vaccines re-prioritize the immune repertoire to more relevant T cells against the novel pathogen. In the context of the COVID-19 pandemic, cellular immune response mediated by CD8+ T cells specific to cross-reactive epitopes from common coronaviruses has been reported as one of the main determinants of immunological protection in SARS-CoV-2 infection (66). Taken together, these findings raise the possibility that individuals vaccinated with BCG present a pre-existing pool of cross-reactive CD8 T cells, which can be recruited during SARS-CoV-2 natural infection or COVID-19 vaccine, resulting in an enhanced cellular immunity.

Several studies have described a protective effect of BCG vaccination against viral infections (11, 15, 26, 67, 68). In fact, it has been suggested that the BCG vaccination program could be associated with a reduction in COVID-19 cases in some countries (69). In this context, the search for sequence similarity between SARS-CoV-2 peptides and peptides from the BCG vaccine has been the main tool available for the identification of potential targets for T cell cross-reactivity, which could be the basis for a BCG-induced heterologous immunity. In a recent study, Eggenhuizen and collaborators (36) used an *in vitro* co-culture system to identify human T cells specific to a BCG-derived peptide, which cross-react with a highly similar peptide from SARS-CoV-2.

Here we present a large-scale computational screening for potential targets of T cell cross-reactivity between SARS-CoV-2 and BCG that might help to explain the possible protective effect of BCG vaccination against COVID-19. We combined standard immunoinformatics methods (e.g., sequence-based HLA-binding prediction) with innovative structural approaches to better account for other aspects of biochemical and structural features that are potential drivers of T cell cross-reactivity. Some of the pHLA comparison methods used here have been proposed and applied in previous studies (39, 48), but have never been deployed to the scale of the analysis described here. The size of this analysis, encompassing the entire proteome of both pathogens of interest, created a series of technical challenges that could not be addressed with off-the-shelf solutions. For instance, our structural analysis included the modeling of 358,386 pHLA complexes with APE-Gen. This is the largest dataset of pHLA structures ever produced, and such large-scale modeling was enabled by the development of a dispatcher to optimize the use of APE-Gen in High Performance Computing clusters. As part of our analysis, we performed the TCR-interacting surface comparison of all possible pairs of pHLAs for a given HLA-I allele, considering 46 regions of interest in each surface, and multiple data points for each region. This was only possible through the implementation of a plugin for image processing with ImageJ. All APE-Gen models produced in this project, as well as the APE-Gen dispatcher and the ImageJ plugin will be made publicly available as part of this study.

Our analysis produced a large dataset of putative cross-reactive clusters involving BCG- and SARS-CoV-2-derived peptides. Then, we explored both qualitative and quantitative approaches to prioritize the most promising pairs of peptides in this dataset. Our initial focus on SARS-CoV-2 peptides with experimentally-determined immunogenicity led to the identification of 40 putative 9-mer peptide pairs with potential cross-reactivity with BCG peptides. This list included the HLA-B*40:01-restricted SARS-CoV-2-derived peptides GEAANFCAL, GEVITFDNL, and FIAGLIAIV which have been independently shown to induce T cell response, INF-γ production, and proliferation in COVID-19 patients (66, 70). In addition, multiple peptides from our top 40 list (**Table 1**) have been reported to induce T cell activation in recent studies analyzing aspects of cellular immunity in COVID-19 patients (71–74). Some of the peptides described by Eggenhuizen and colleagues as cross-reactive between BCG and SARS were found in the top 16,000 putative SARS-BCG cross-reactive peptide pairs (**Supplementary Table 2**) (36).

In our analysis, the number of putative clusters of cross-reactive peptides varied among HLA-I alleles. Although most of the peptides were predicted to bind to HLA-A*01:01, most of the cross-reactive clusters were restricted to HLA-B*15:03. Note that the importance of the HLA-B*15:03 allele for the presentation of SARS-CoV-2-derived peptides has already been described in previous work. For instance, molecular docking studies performed by Albagi et al. indicated higher affinity of Spike-derived peptide for the HLA-B*15:03 allele (75). In another study, Barquera and colleagues analyzed the binding affinities of peptides derived from complete proteomes of seven pandemic human viruses (including coronaviruses), against 438 Class I and Class II HLA proteins. In their study, statistical modeling indicated that HLA-B*15:03 binds to more than 200 peptides with strong affinity, with only a minimum number of peptides being predicted as weak/non-binders for this allele (76). Finally, a comprehensive *in silico* study by Nguyen and colleagues suggested that HLA-B*15:03 can generate cross-protective T cell dependent immunity, due to a greater ability to present highly conserved SARS-CoV-2 peptides (77). Our study complements these previous findings by incorporating large-scale structural modeling and structure-based comparison of pHLA complexes on the identification of potential targets of T cell cross-reactivity. Taken together, these findings further corroborate the notion that differences in HLA-I genotypes can alter the course of the COVID-19 disease and its transmission (78), and that HLA-B*15:03 might contribute to increased cellular immunity against SARS-CoV-2. On the other hand, the allele with the least number of predicted clusters in our analysis was HLA-B*51:01 (**Figure 2C**), which could suggest an increased susceptibility to COVID-19. In fact, a study on genetic association with susceptibility to SARS-CoV-2 infection and disease severity identified HLA-B*51:01, as well as HLA-A*11:01 and HLA-C*14:02, as the most prevalent in patients with severe disease (79).

The response mediated by CD4+ T cells is also important during SARS-CoV-2 infection and the vaccine immunity (80, 81). Although this was not the major goal of our study, we ran a sequence-based predictor on the selected 40 possible cross-reactive BCG-SARS pairs to analyze their capacity to bind HLA class II molecules. We have found only one epitope (YLASGGQPI) that can strongly bind to the human HLA-DRB1*07:01 (data not shown).

To further explore our dataset, going beyond available experimental data, we performed a quantitative analysis to identify both (i) clusters with higher density neighborhoods of potentially cross-reactive BCG pHLA-targets, and (ii) clusters with SARS epitopes that were shared across different HLAs (**Figures 1** and **3**). This effort identified a list of SARS peptides representing the 50 higher-density neighborhoods. These candidates are interesting because they could indicate instances in which T cell cross-reactivity with a single SARS-CoV-2 peptide could be primed by multiple BCG peptides, increasing its potential involvement in heterologous immunity. In addition, our data could help the analysis of results from several ongoing clinical trials on the protective effect of BCG vaccination for COVID-19. One limitation of our study is the lack of experimental validation of these peptides. These targets should be further investigated and confirmed in future studies.

Finally, we also identified clusters of potential cross-reactive targets that were shared across multiple HLA-I alleles. Considering the vast diversity of HLA-I alleles in the human population (i.e., over 19,000 alleles), HLA-I restriction is a concern for vaccine development. Even a highly immunogenic SARS-CoV-2 peptide will not constitute a useful target for vaccine development if its presentation to T cells is restricted to an HLA-I allele that is rare in the human population. On the other hand, peptides displayed by prevalent HLA-I alleles, or even multiple HLAs, would make very interesting targets for vaccine development due to broader population coverage. In fact, we have found two peptides - FSAVGNICY and QPGQTFSVL - that were (i) included in our top 50 high-density neighborhoods, (ii) can be presented by multiple alleles, and (iii) are immunogenic targets, with the capacity to elicitate CD8+ T cell response.

In the context of the current pandemic it is important to note that despite ongoing success with multiple COVID-19 vaccines, there are still open questions and concerns regarding the rise of different SARS-CoV-2 variants. In addition, other coronaviruses represent a continuous threat of new pandemics, considering the existence of large natural reservoirs (82). In this context, the development of peptide-based vaccines targeting conserved regions of coronaviruses, and presenting cross-reactivity with existing pools of memory T cells in the population, could be an interesting strategy to complement and extend the protection conferred by existing COVID-19 vaccines (83–85). We hope our work can contribute to these efforts by suggesting high priority targets for potential T cell cross-reactivity with BCG-derived peptides.

## Data Availability Statement

The raw data supporting the conclusions of this article will be made available by the authors, without undue reservation.

## Author Contributions

RT: Conceptualization, Software, Methodology, Validation, Investigation, Data Curation, Writing, Visualization. MM: Conceptualization, Software, Methodology, Validation, Investigation, Data Curation, Writing, Visualization. AF: Methodology, Software, Validation, Formal analysis, Investigation, Data Curation, Writing, Visualization. FR: Methodology, Software, Validation, Formal analysis, Data Curation. RB: Methodology, Software, Validation, Formal analysis, Data Curation. LK: Resources, Writing, Supervision, Project administration, Funding acquisition. TF: Methodology, Software, Validation, Formal analysis, Resources, Data Curation, Writing, Supervision, Project administration, Funding acquisition. DA: Conceptualization, Methodology, Resources, Writing, Supervision, Project administration, Funding acquisition. AS: Conceptualization, Methodology, Resources, Writing, Supervision, Project administration, Funding acquisition. All authors contributed to the article and approved the submitted version.

## Funding

MM was supported by a CPRIT fellowship on RP170593. MM and LK were also supported in part by NCI 1U01CA258512-01 and NSF DBI 2033262. RT and AS were financed in part by the Coordenação de Aperfeiçoamento de Pessoal de Nivel Superior – Brasil (CAPES) – Finance Code 001. AF and DA were supported in part by funds from the University of Houston. AS was also supported by FAPERGS 20/2551-0000258-6.

## Conflict of Interest

The authors declare that the research was conducted in the absence of any commercial or financial relationships that could be construed as a potential conflict of interest.

## Publisher’s Note

All claims expressed in this article are solely those of the authors and do not necessarily represent those of their affiliated organizations, or those of the publisher, the editors and the reviewers. Any product that may be evaluated in this article, or claim that may be made by its manufacturer, is not guaranteed or endorsed by the publisher.
