# Supplementary figures and images for: Large-Scale Structure-Based Screening of Potential T Cell Cross-Reactivities Involving Peptide-Targets From BCG Vaccine and SARS-CoV-2
Source: Front Immunol. 2022 Jan 13;12:812176. doi: 10.3389/fimmu.2021.812176 (PMC8793865; doi:10.3389/fimmu.2021.812176)

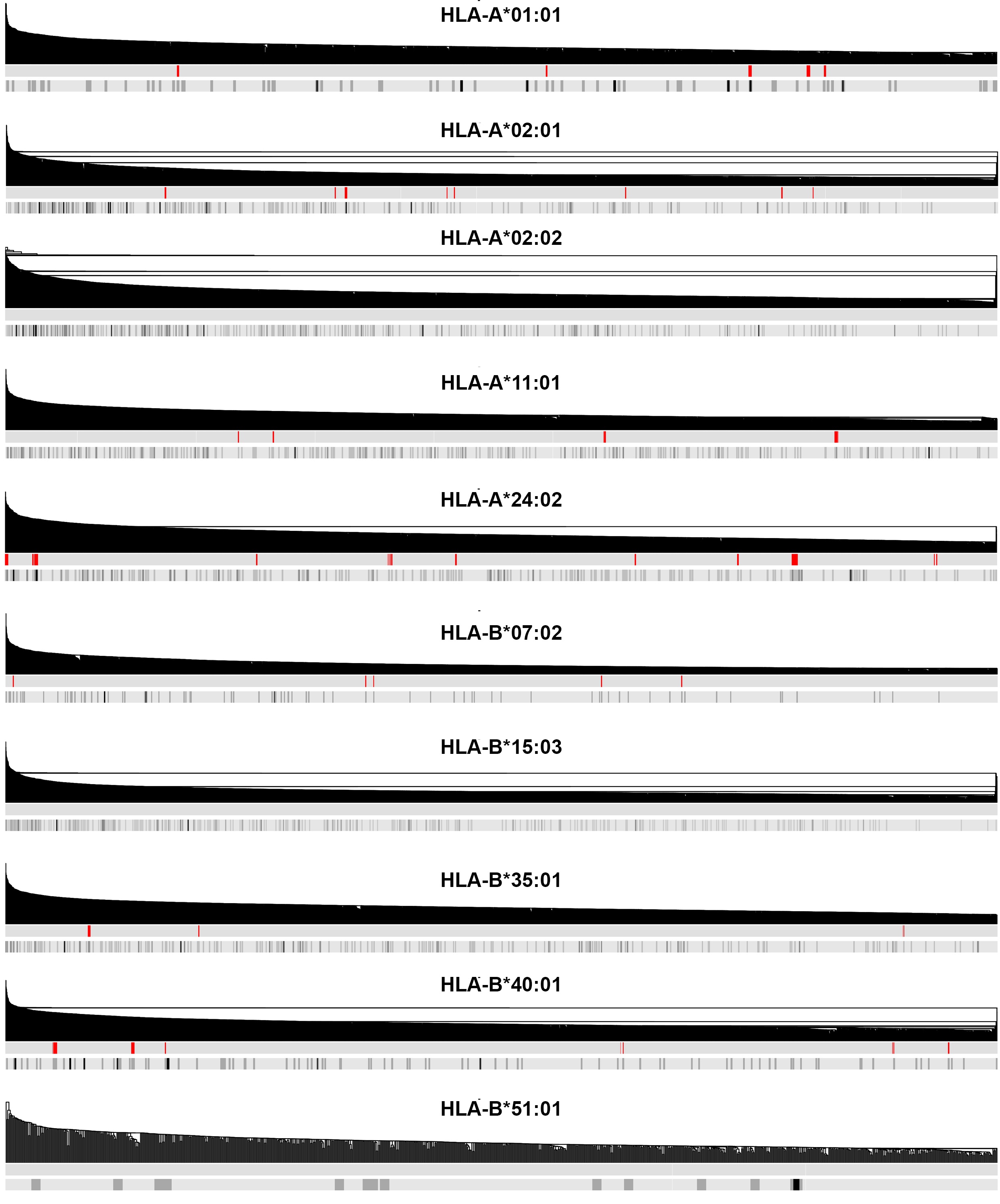

Supplement: Supplementary file 1 [file Image_1.jpeg]

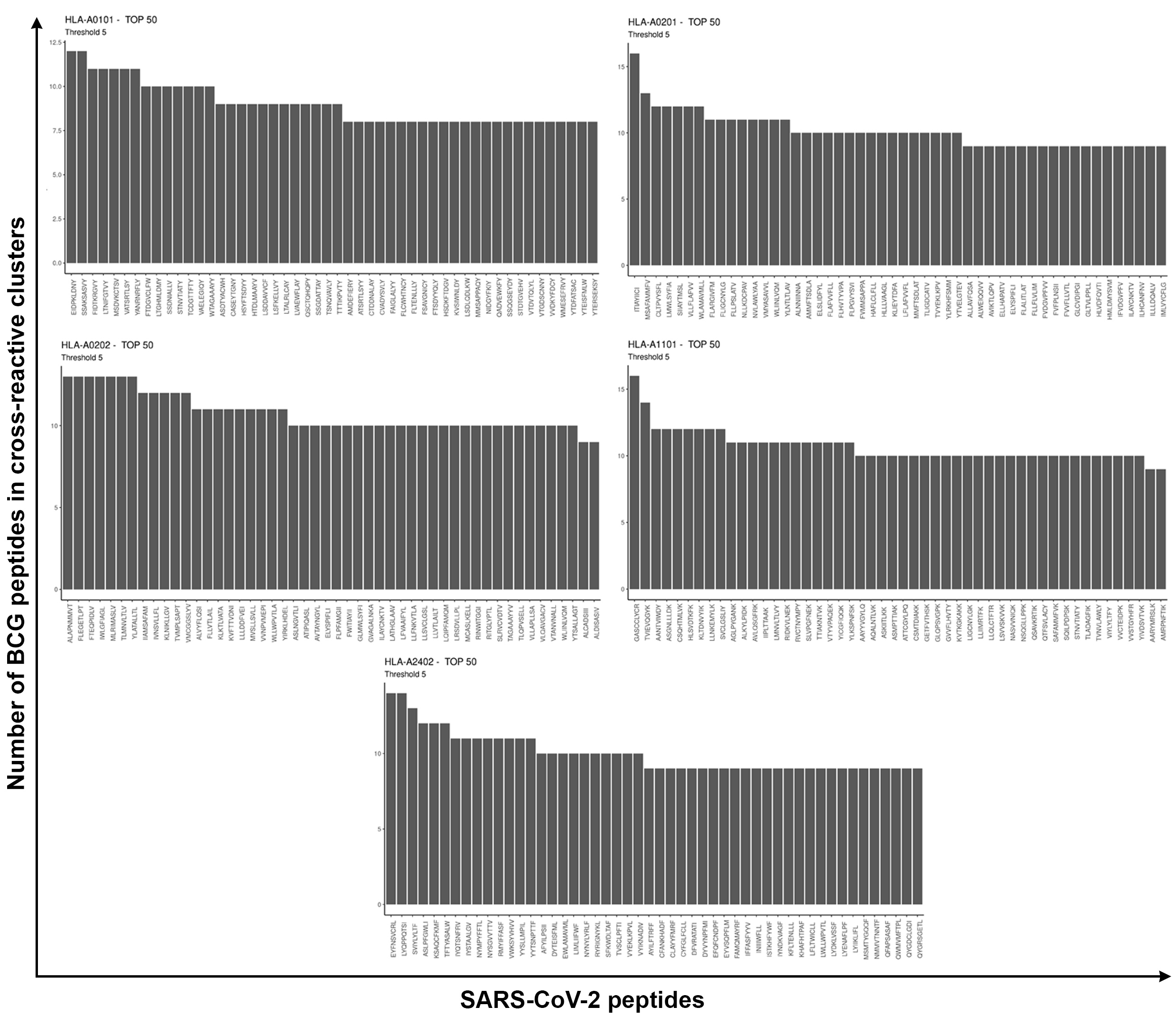

Supplement: Supplementary file 2 [file Image_2.jpeg]

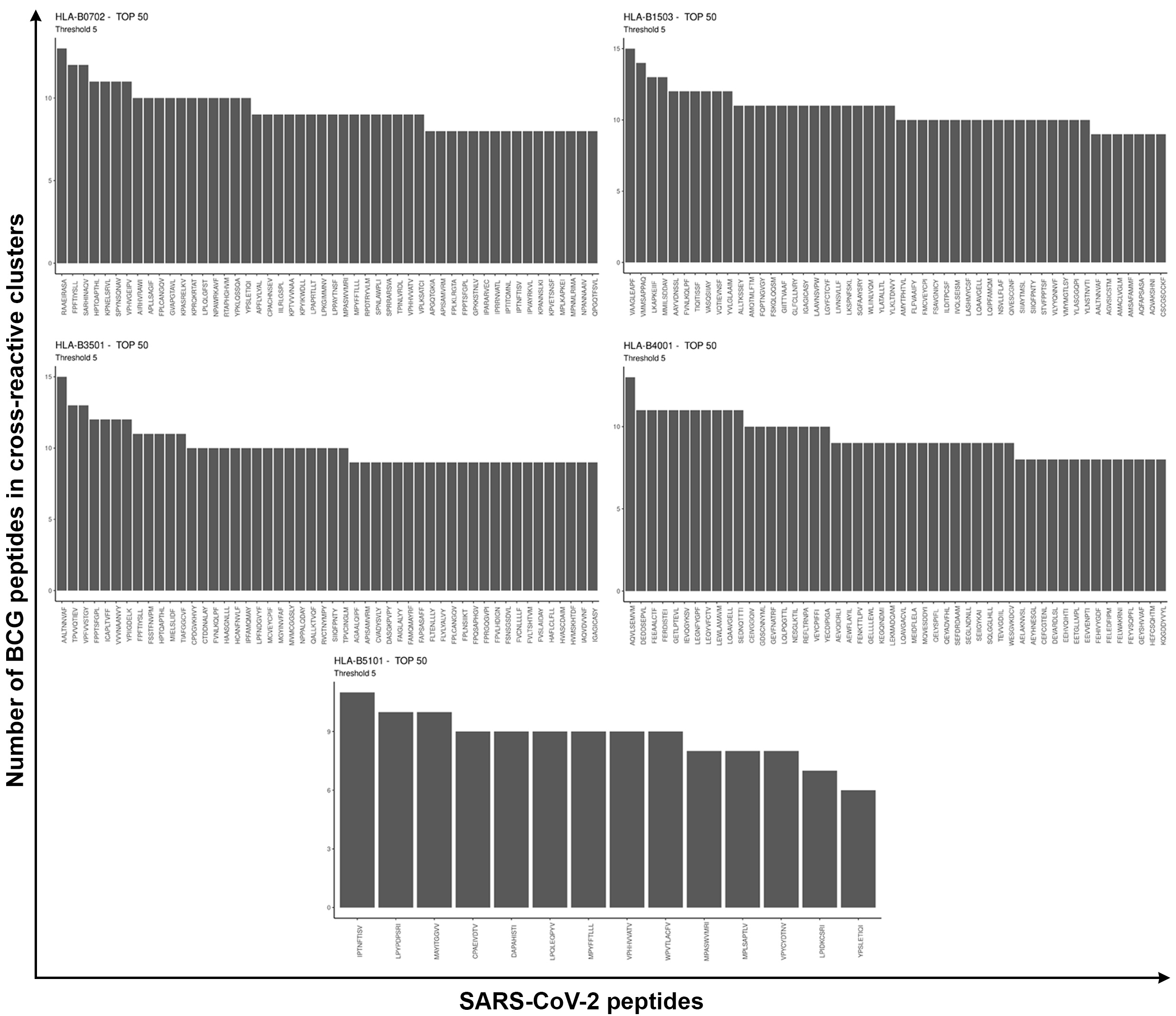

Supplement: Supplementary file 3 [file Image_3.jpeg]
