# Supplementary material for: Large-Scale Structure-Based Screening of Potential T Cell Cross-Reactivities Involving Peptide-Targets From BCG Vaccine and SARS-CoV-2
Source: Front Immunol. 2022 Jan 13;12:812176. doi: 10.3389/fimmu.2021.812176 (PMC8793865; doi:10.3389/fimmu.2021.812176)
Supplement: Supplementary file 4 [file Image_4.pdf]

PAIR #1

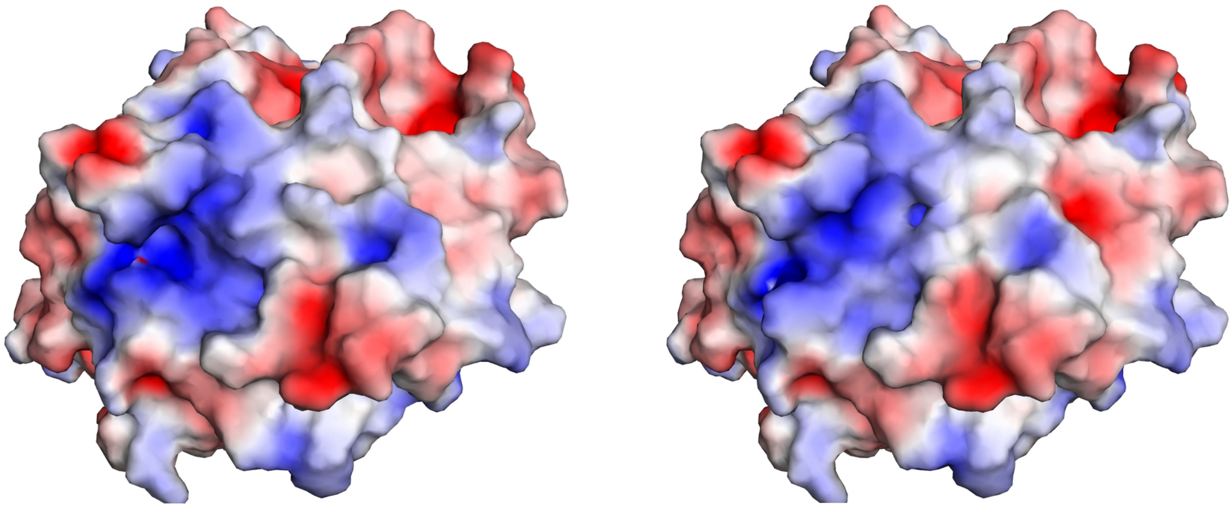

HLA-A0101-SARS_CNDPFLGVY

HLA-A0101-BCG_MTAFGVEPY

PAIR #2

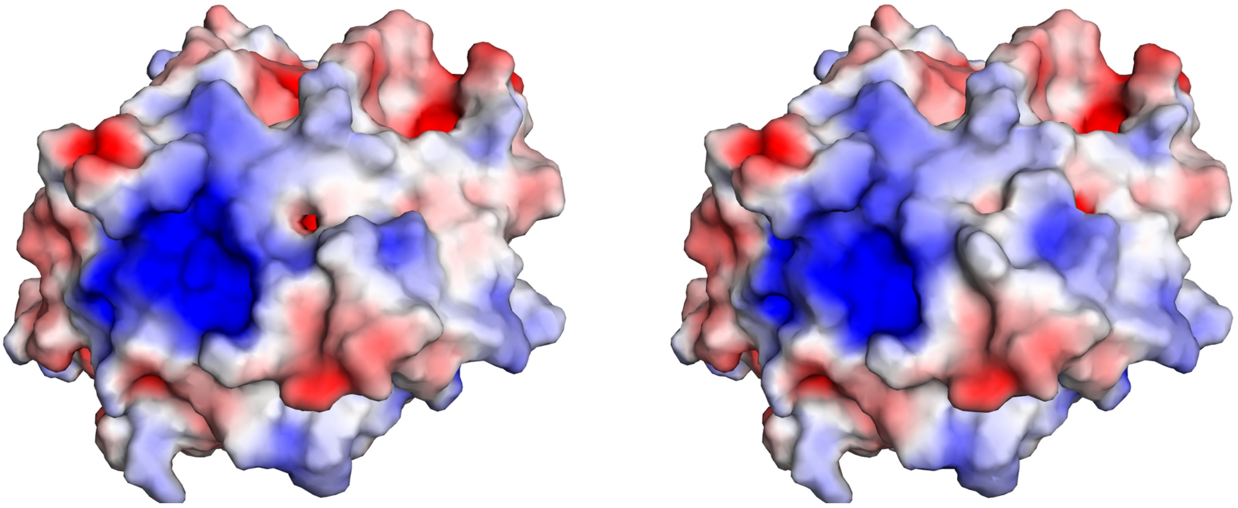

HLA-A0101-SARS_FSAVGNICY

HLA-A0101-BCG_WTDVKFALI

PAIR #3

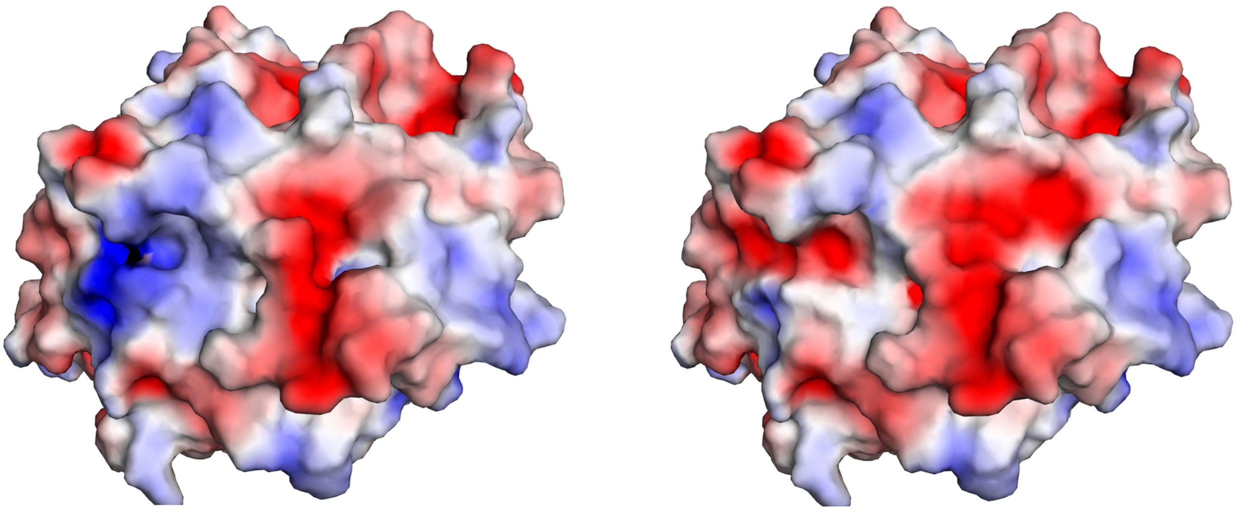

HLA-A0101-SARS_GTDLEGNFY

HLA-A0101-BCG_EVDSAFDGY

PAIR #4

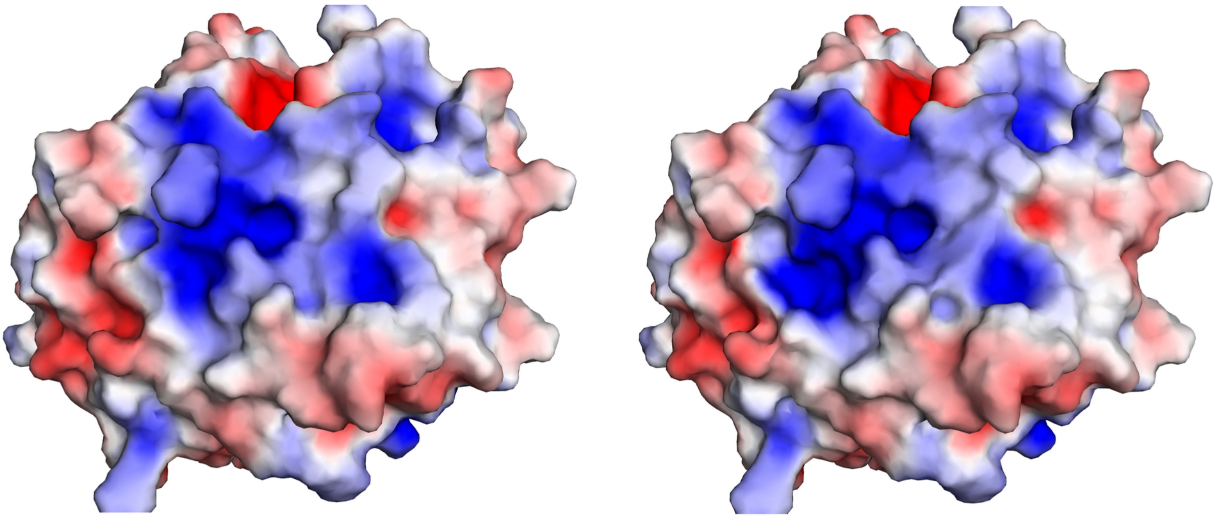

HLA-A0201-SARS_FIAGLIAIV HLA-A0201-BCG_TLAGLLPPV

PAIR #5

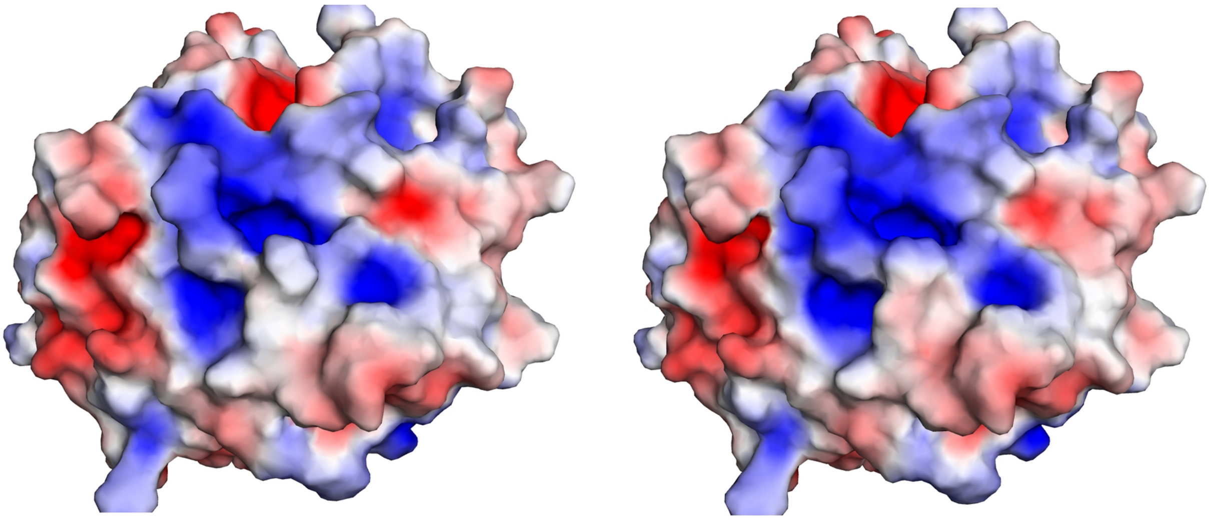

HLA-A0201-SARS_FVFLVLLPL HLA-A0201-BCG_WVFLVNLPL

PAIR #6

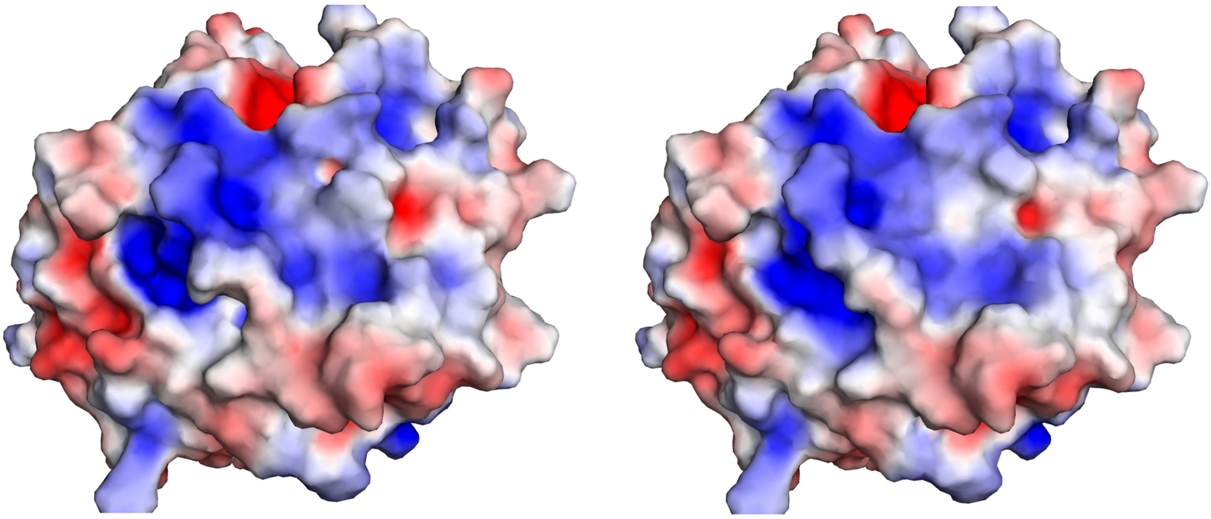

HLA-A0201-SARS_GLMWLSYFI HLA-A0201-BCG_TMWLHVPAV

PAIR #7

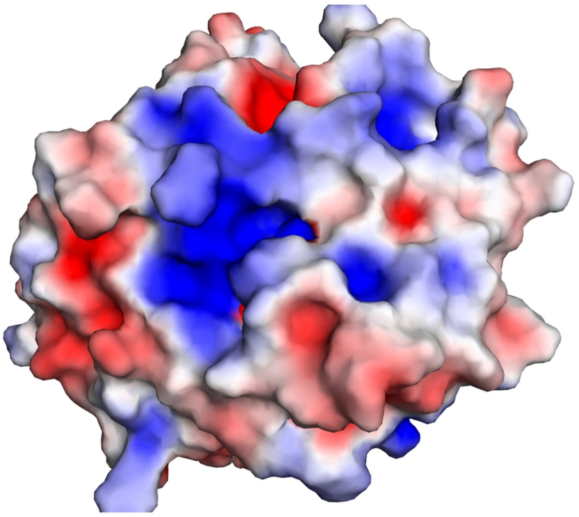

HLA-A0201-SARS_ILGLPTQTV

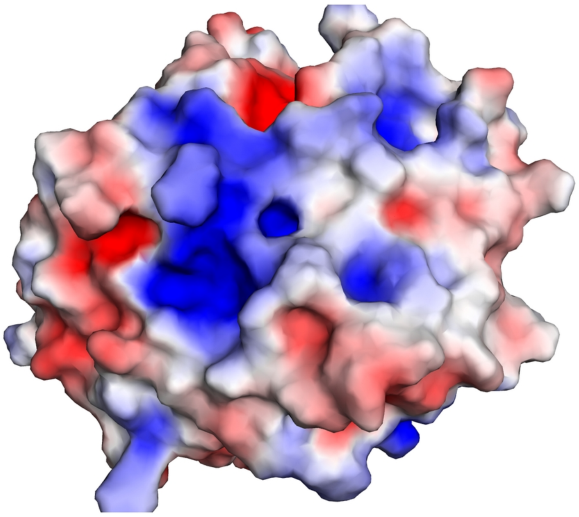

HLA-A0201-BCG_IVAALLVTI

PAIR #8

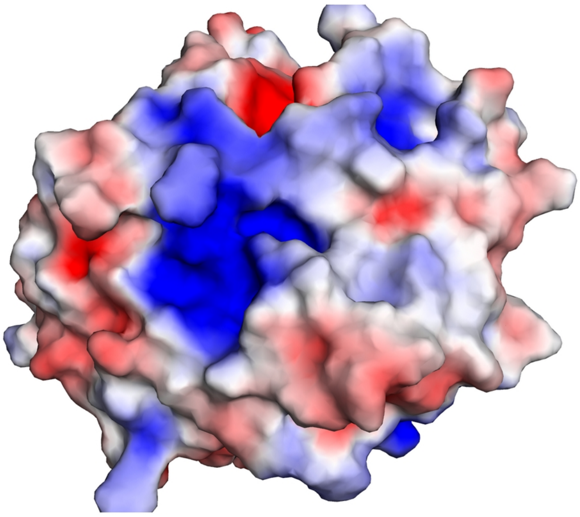

HLA-A0201-SARS_IVAGGIVAI

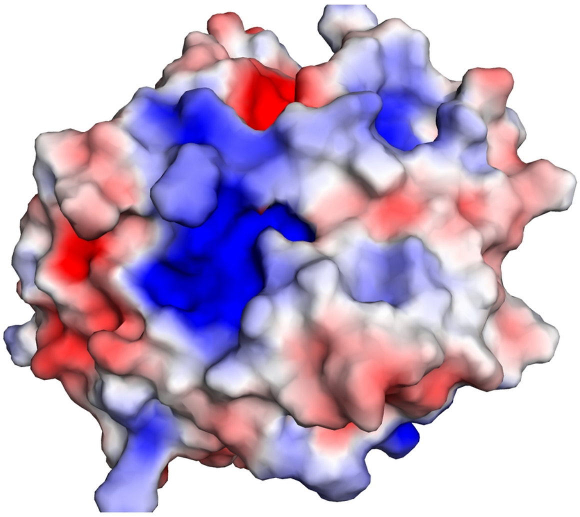

HLA-A0201-BCG_NLTGGIVAL

PAIR #9

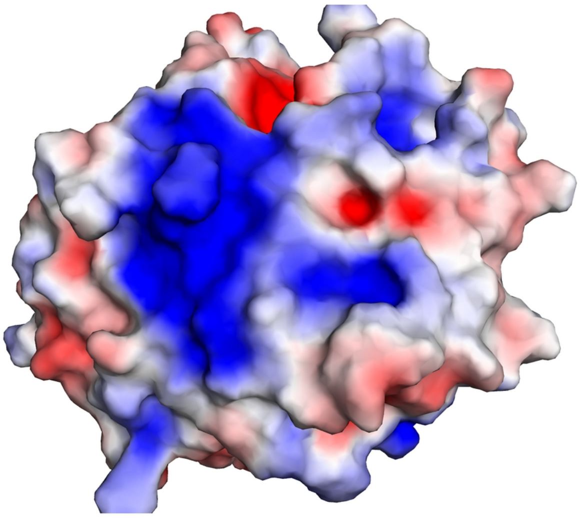

HLA-A0201-SARS_KLVNKFLAL

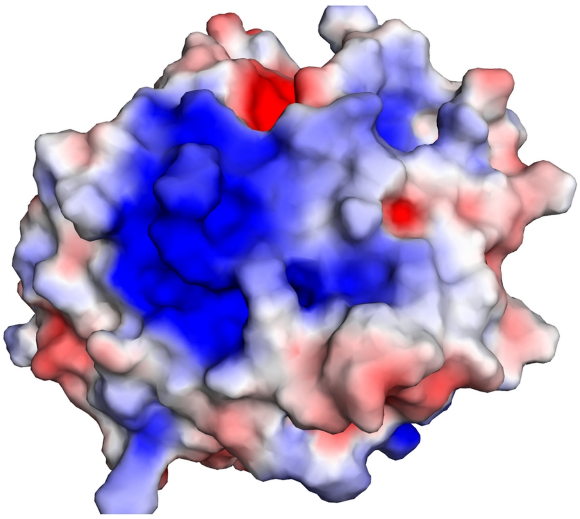

HLA-A0201-BCG_KMAKSVLLA

PAIR #10

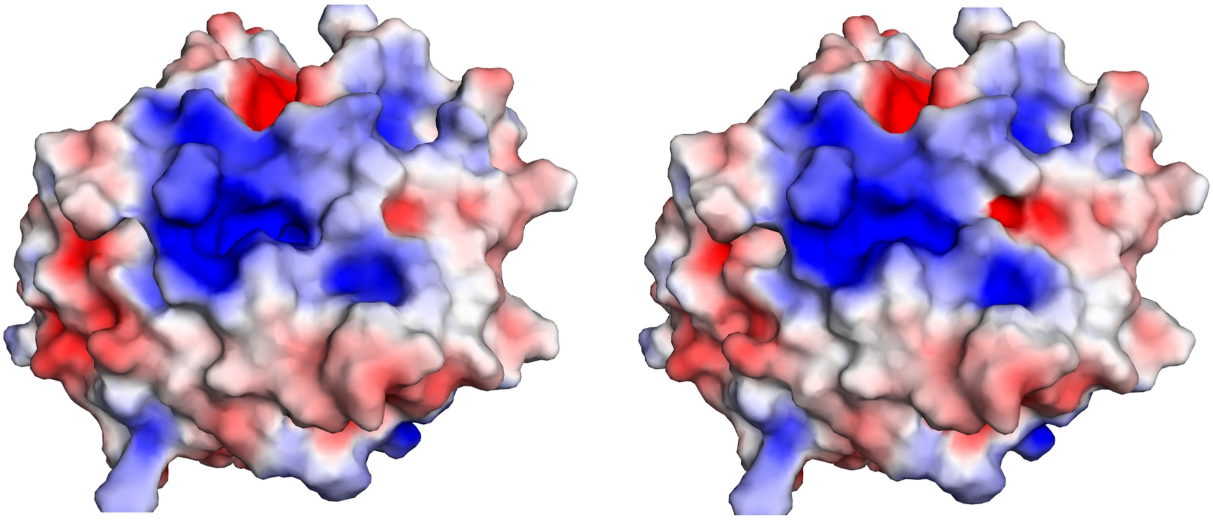

HLA-A0201-SARS_TLMNVLTLV HLA-A0201-BCG_ALVLNLLPI

PAIR #11

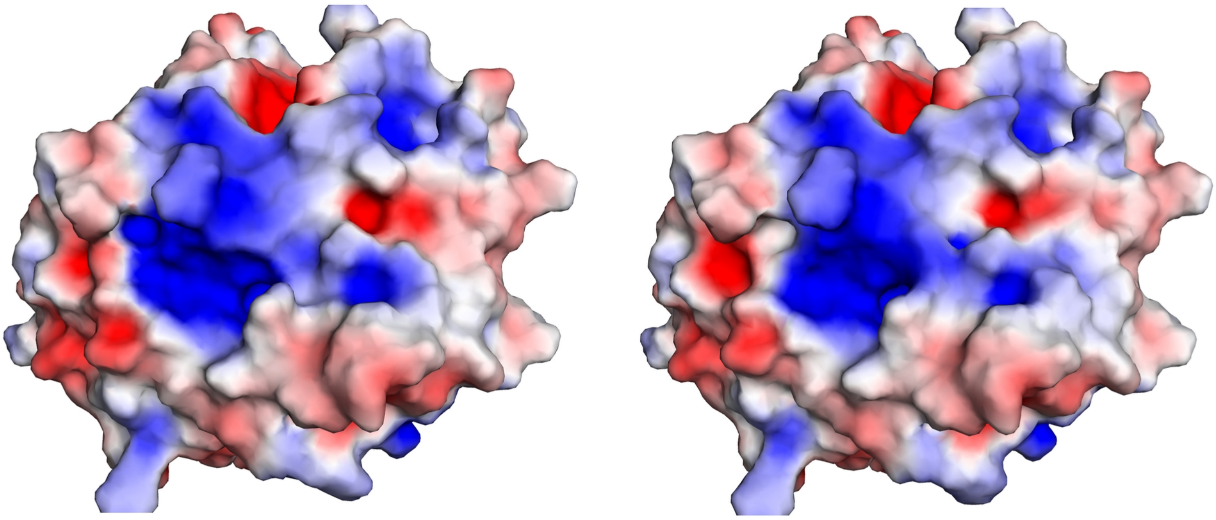

HLA-A0201-SARS_YLASGGQPI HLA-A0201-BCG_LLSAAGVPL

PAIR #12

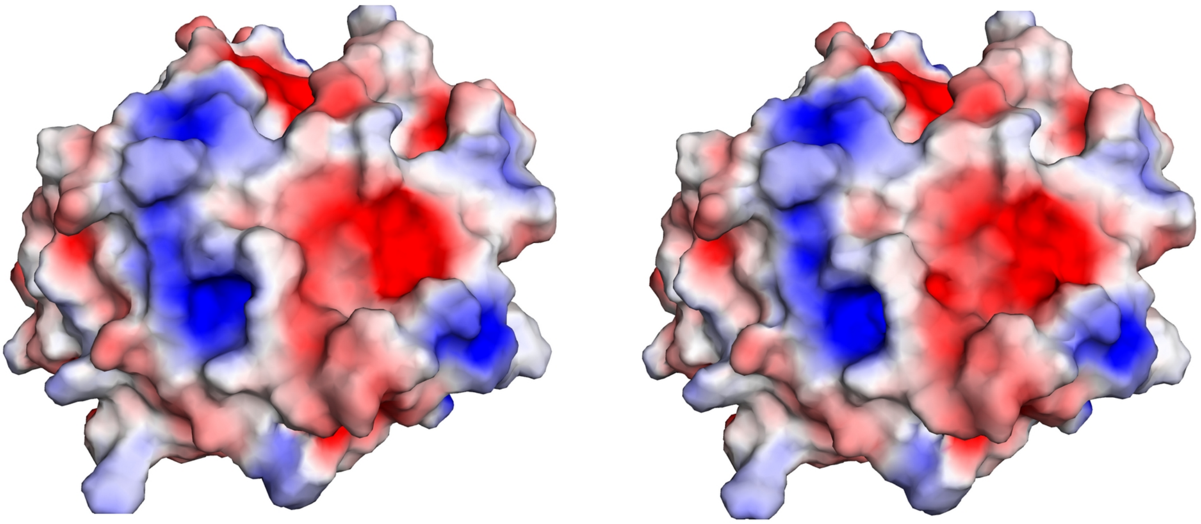

HLA-A1101-SARS_ATVVIGTSK HLA-A1101-BCG_GLNVNTLSY

PAIR #13

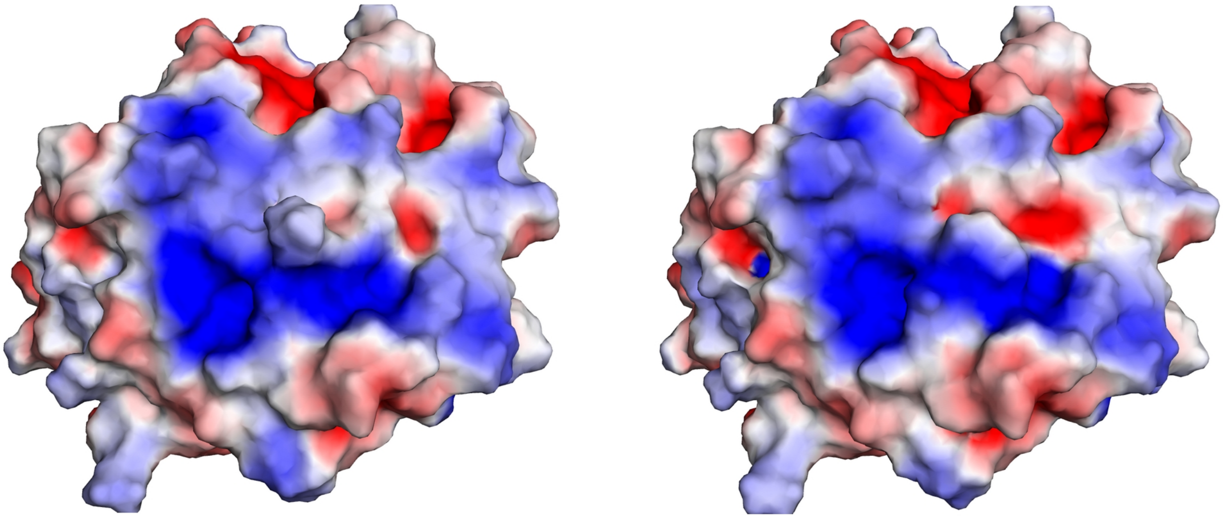

HLA-A1101-SARS_VVNARLRAK HLA-A1101-BCG_IVASRGAQK

PAIR #14

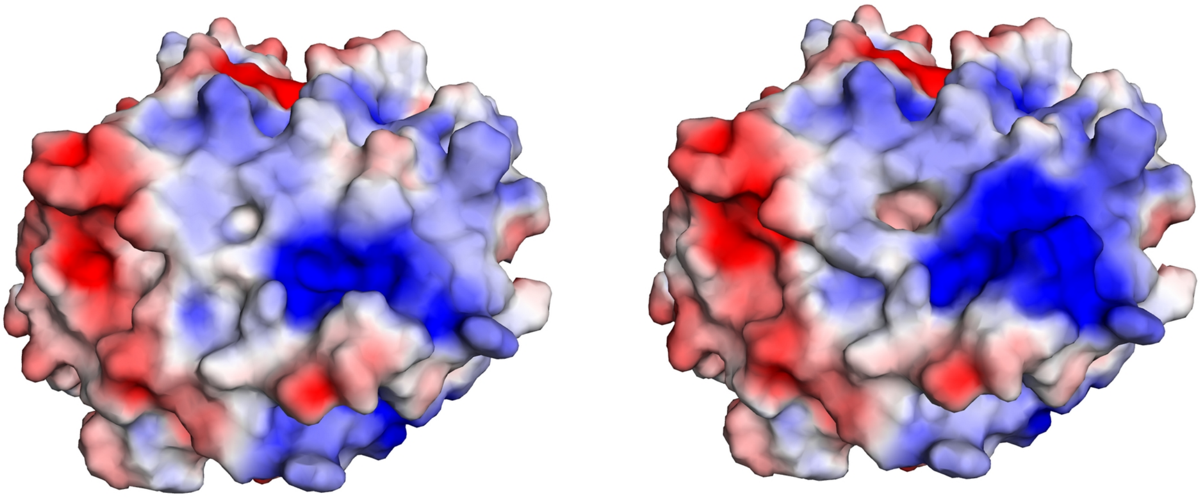

HLA-A2402-SARS_AYANRNRFL HLA-A2402-BCG_VWAQVRNRL

PAIR #15

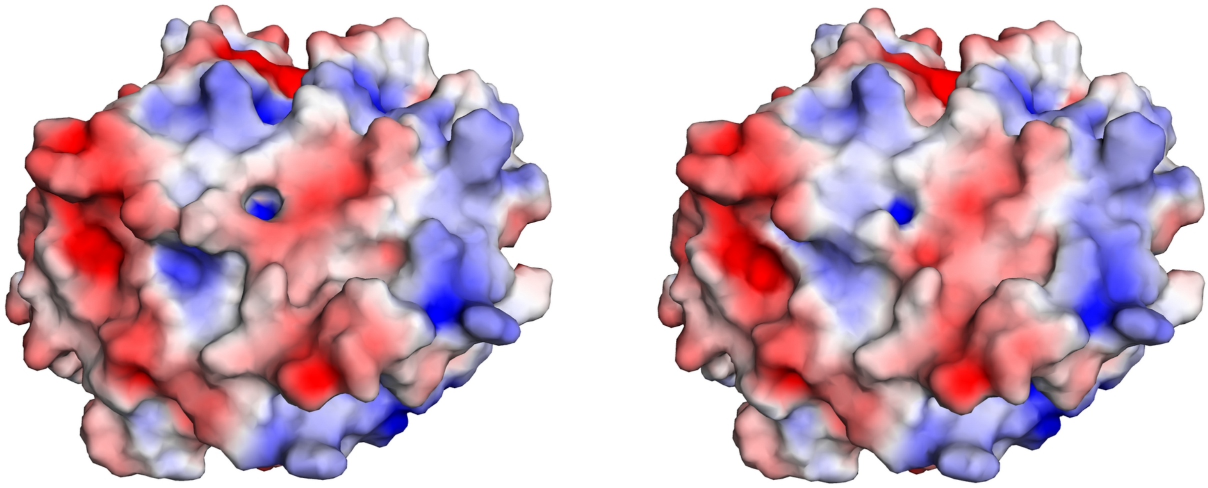

HLA-A2402-SARS_IFFITGNTL HLA-A2402-BCG_TFGALAITL

PAIR #16

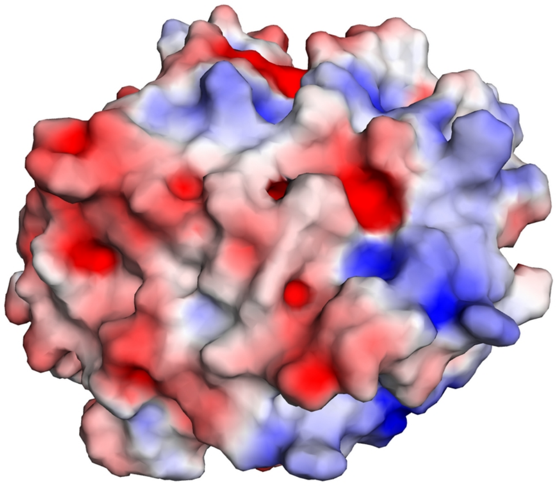

HLA-A2402-SARS_MFTPLVPFW

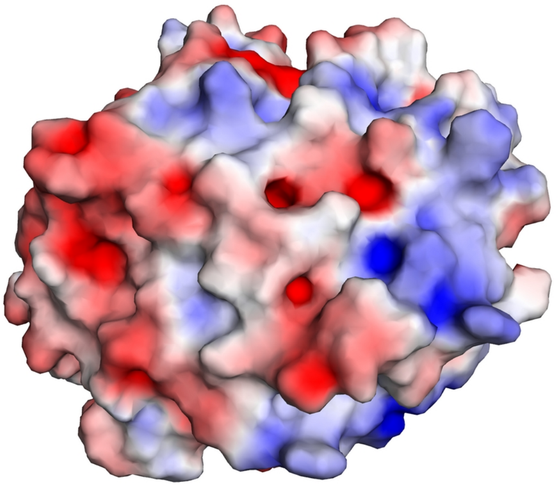

HLA-A2402-BCG_IYPPQVALV

PAIR #17

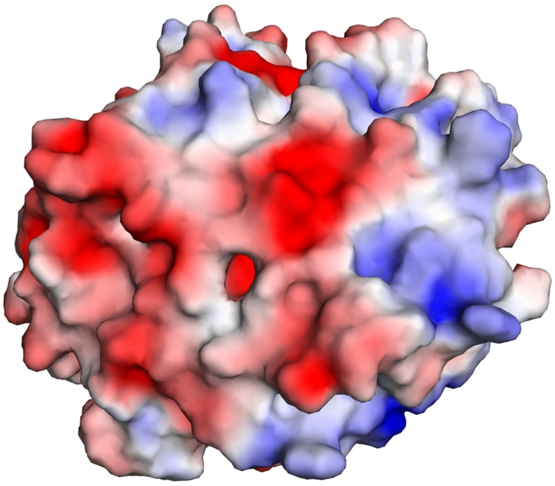

HLA-A2402-SARS_TFNGECPNF

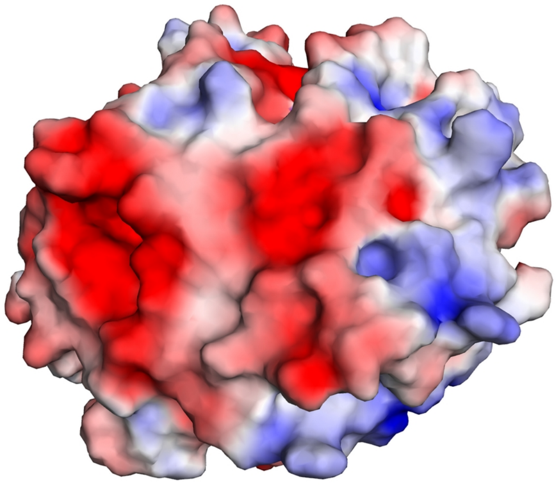

HLA-A2402-BCG_EYLETIHTW

PAIR #18

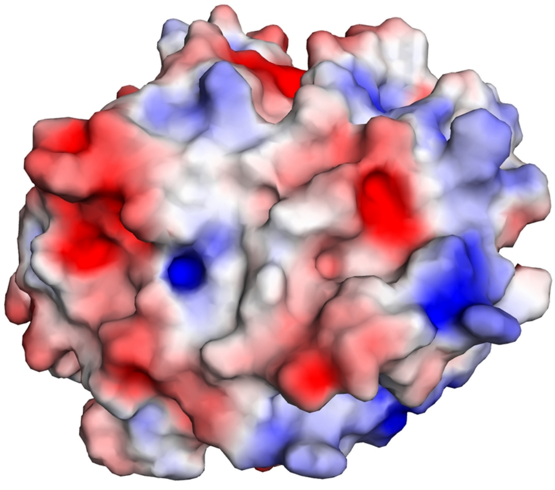

HLA-A2402-SARS_VFVSNGTHW

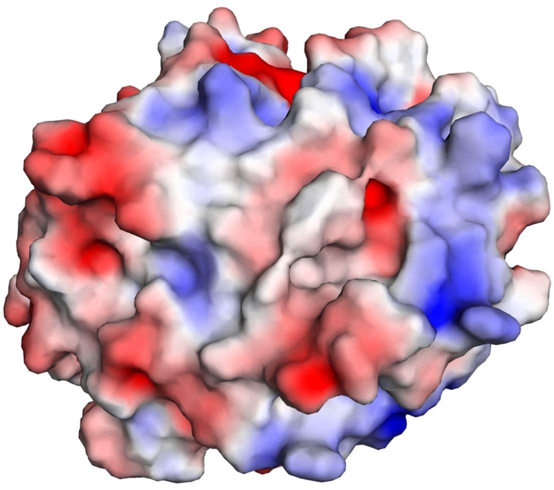

HLA-A2402-BCG_SYIAYAPQL

PAIR #19

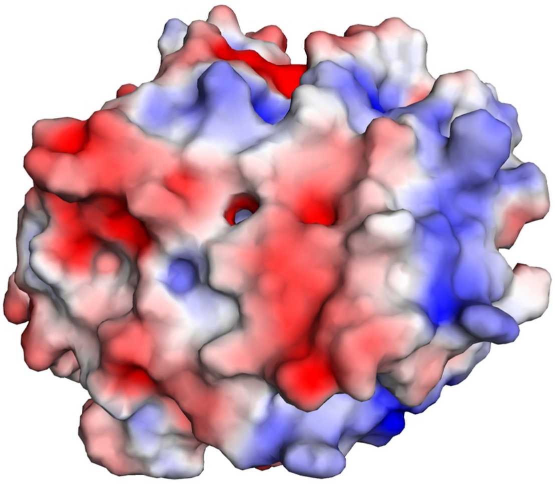

HLA-A2402-SARS_VYMPASWVM

HLA-A2402-BCG_LYGIFIVWL

PAIR #20

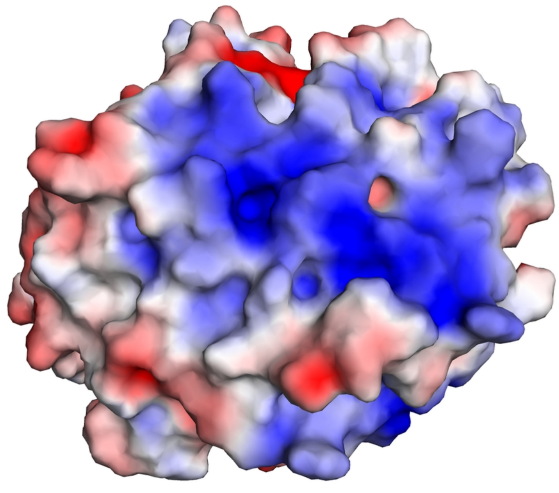

HLA-A2402-SARS_YFMRFRRAF

HLA-A2402-BCG_AWRRLTKVI

PAIR #21

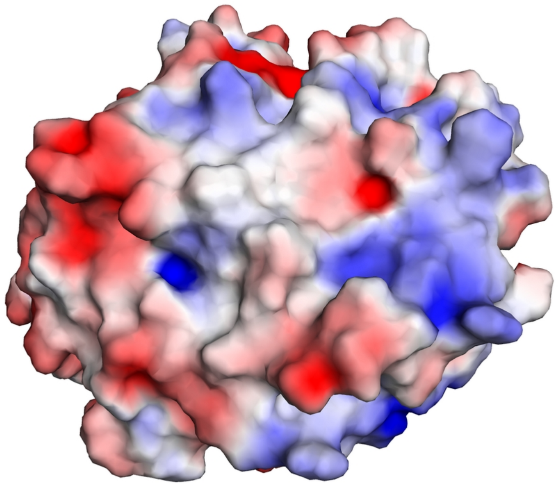

HLA-A2402-SARS_YFPLQSYGF

HLA-A2402-BCG_GWPTWGMIL

PAIR #22

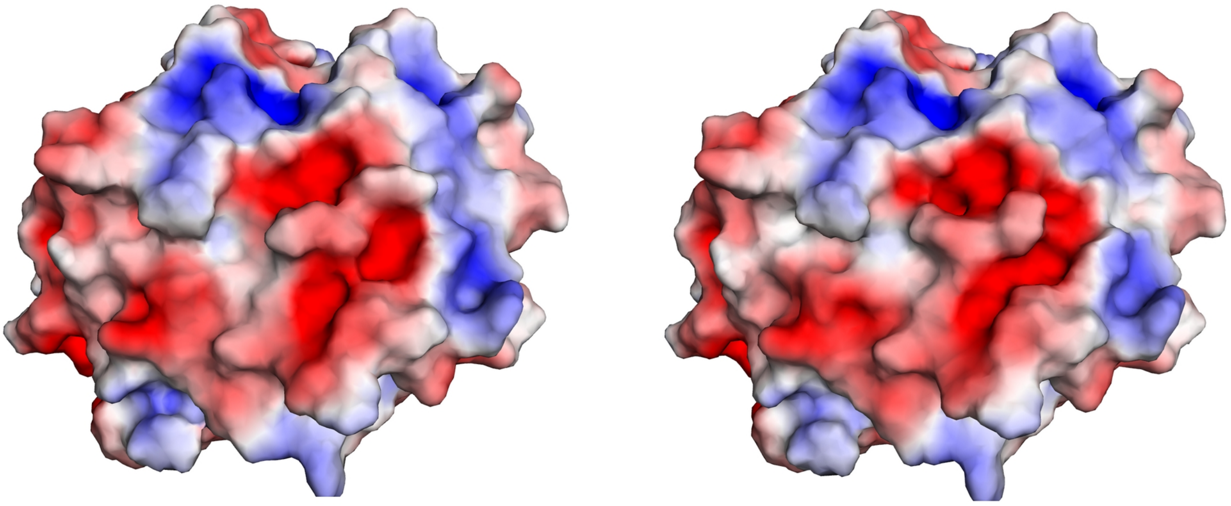

HLA-B0702-SARS_LPNNTASWF

HLA-B0702-BCG_APNSGLVAA

PAIR #23

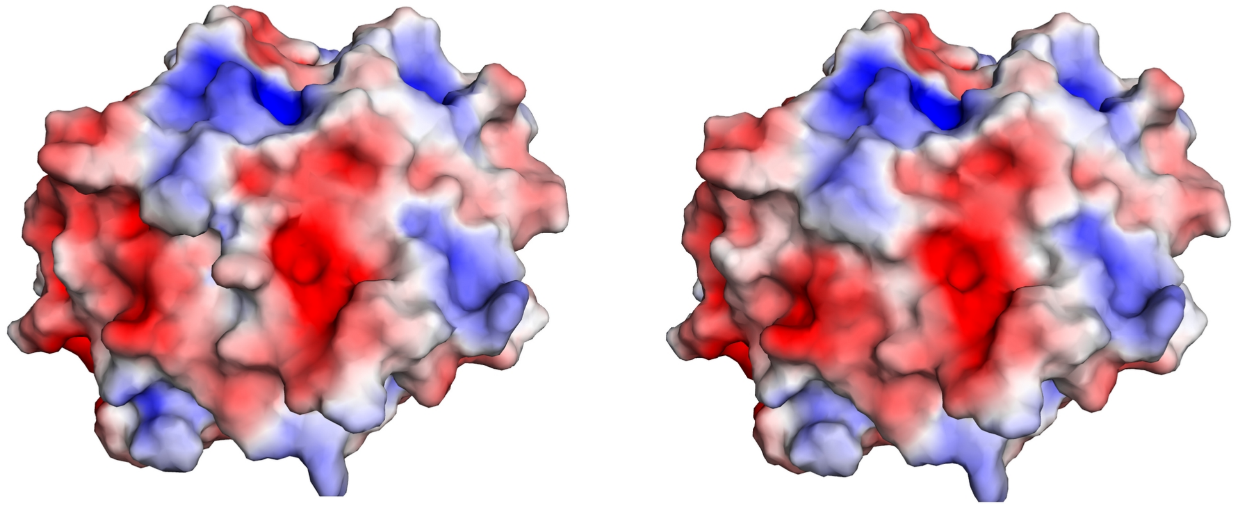

HLA-B0702-SARS_QPGQTFSVL

HLA-B0702-BCG_FPMLQFSLL

PAIR #24

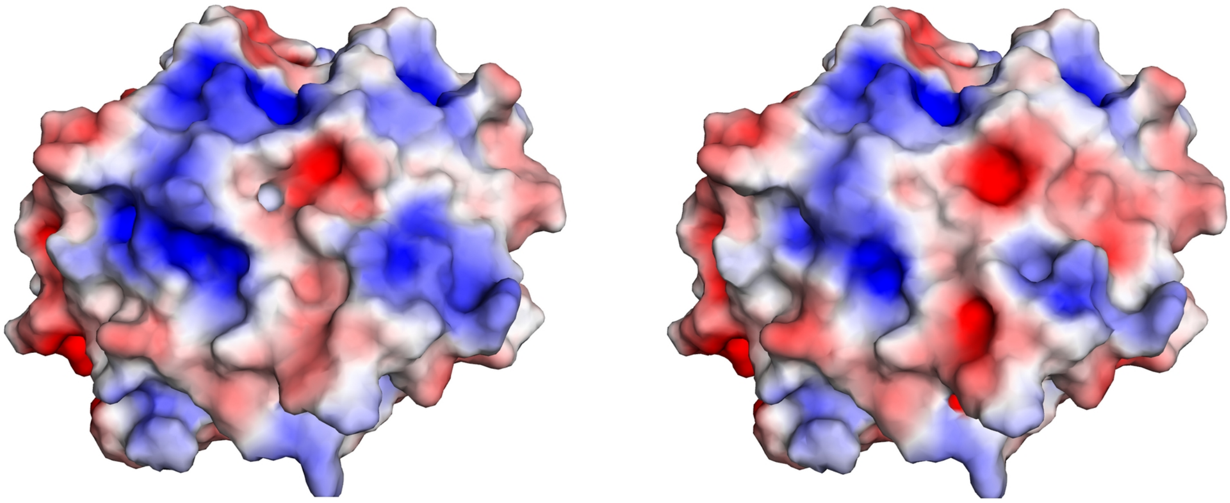

HLA-B0702-SARS_RARSVSPKL

HLA-B0702-BCG_RAATAAMVM

PAIR #25

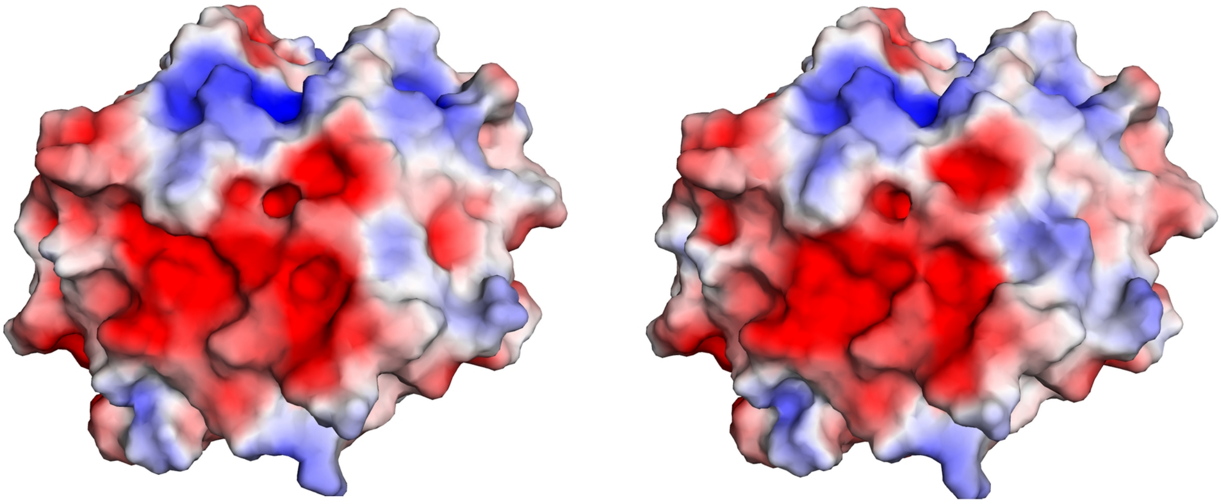

HLA-B0702-SARS_TPRDLGACI HLA-B0702-BCG_LVVDAARAM

PAIR #26

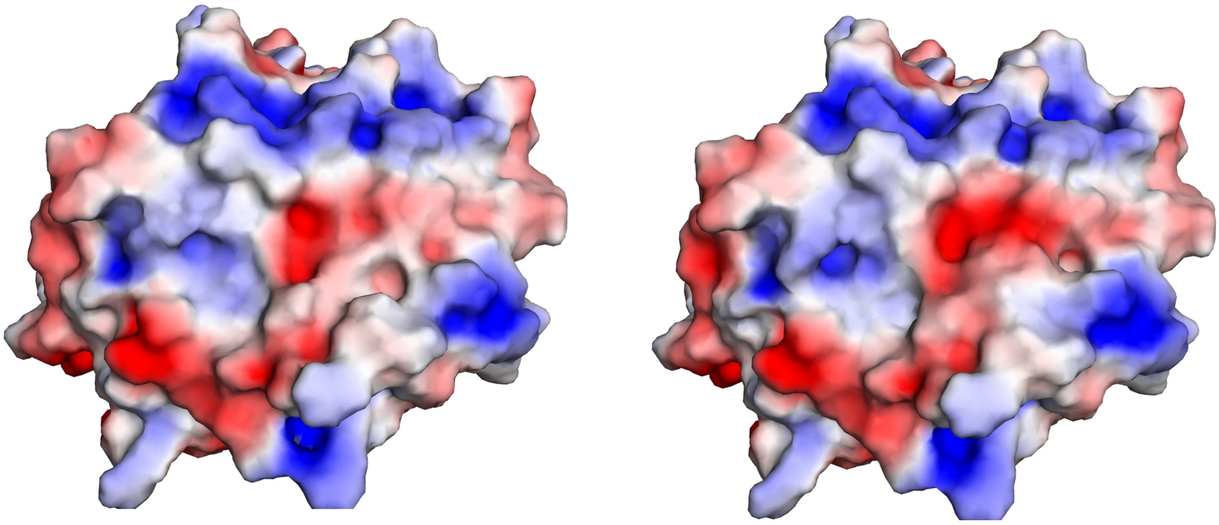

HLA-B4001-SARS_GEAANFCAL HLA-B4001-BCG_TEVLAAQHL

PAIR #27

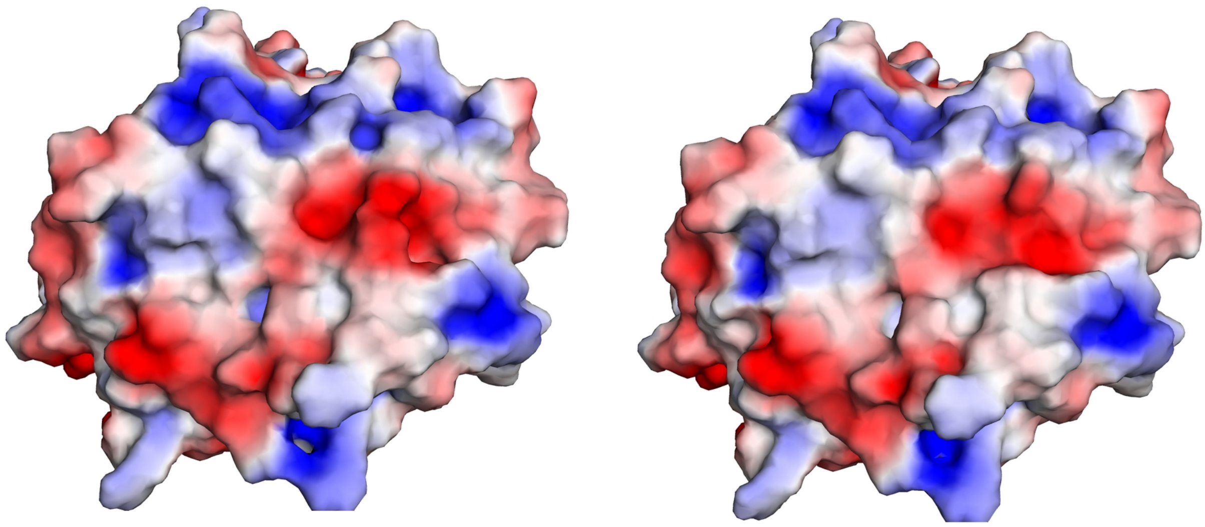

HLA-B4001-SARS_GEVITFDNL HLA-B4001-BCG_SEVVVFDAA

PAIR #28

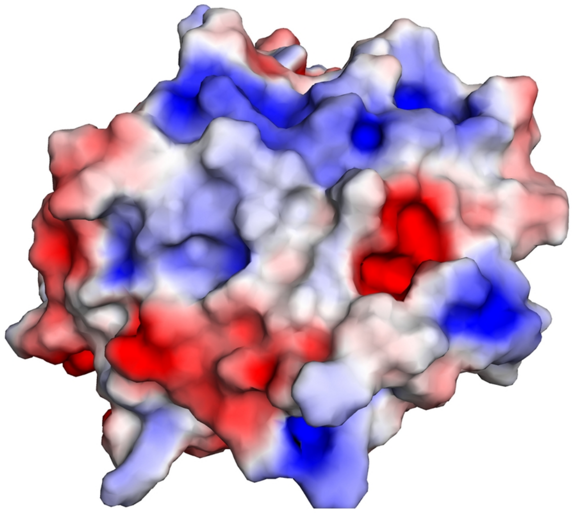

HLA-B4001-SARS_SELLTPLGI

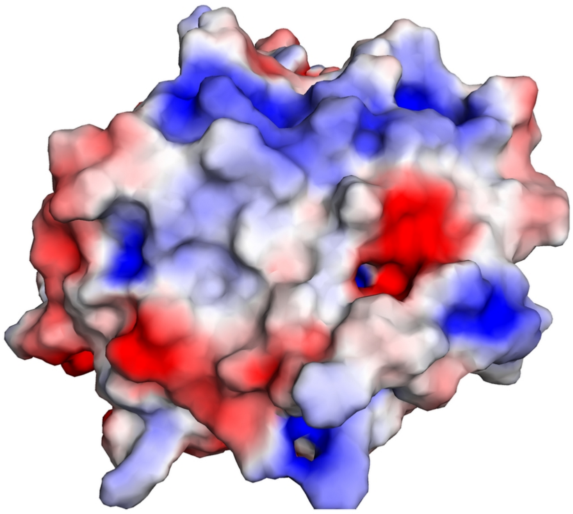

HLA-B4001-BCG_AEMTVALLL

PAIR #29

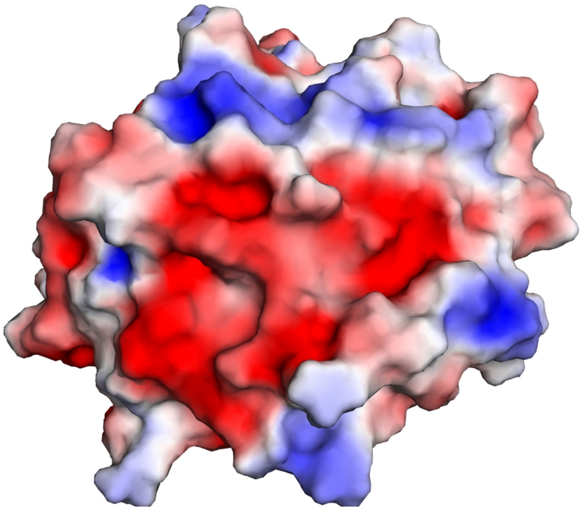

HLA-B4001-SARS_WEPEFYEAM

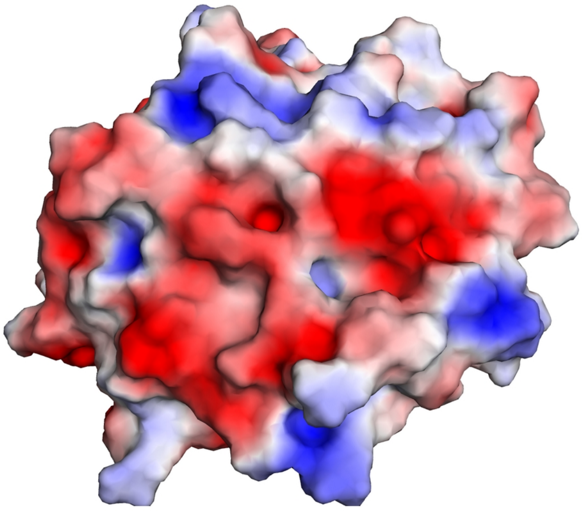

HLA-B4001-BCG_AELEAQQEL

PAIR #30

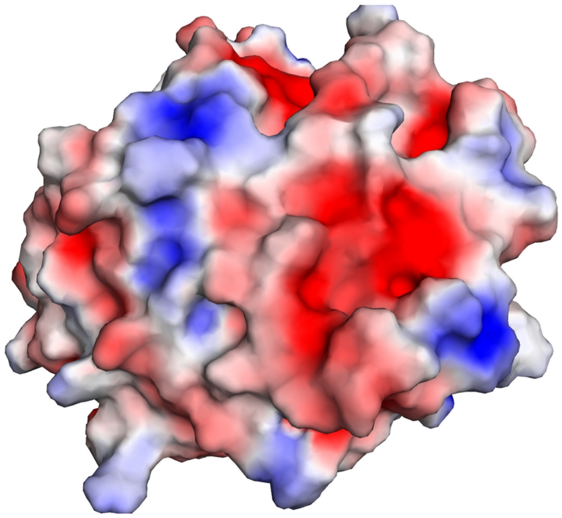

HLA-A1101-SARS_QVVDMSMTY

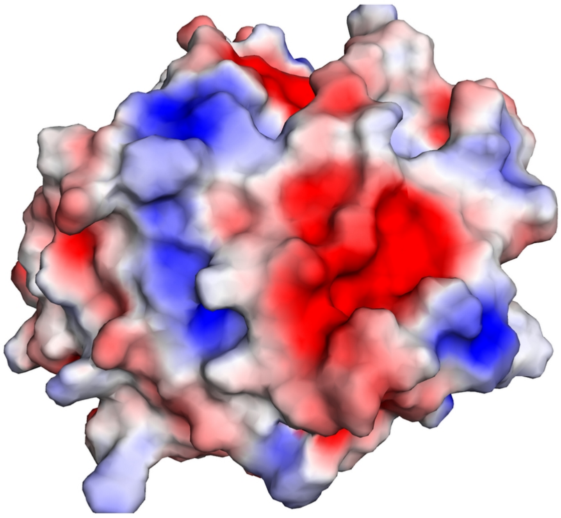

HLA-A1101-BCG_AQPTEPVLK

PAIR #31

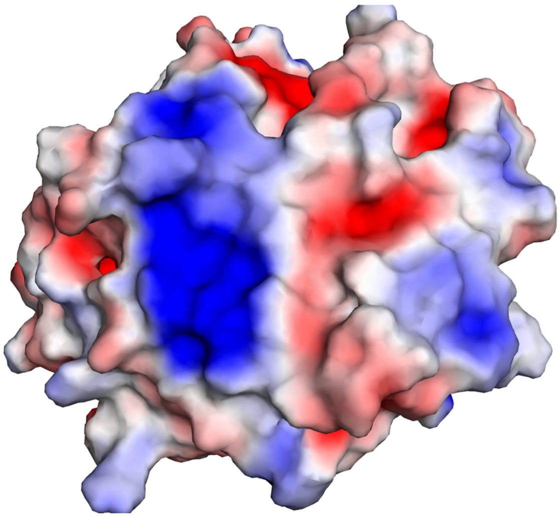

HLA-A1101-SARS_SASKIITLK

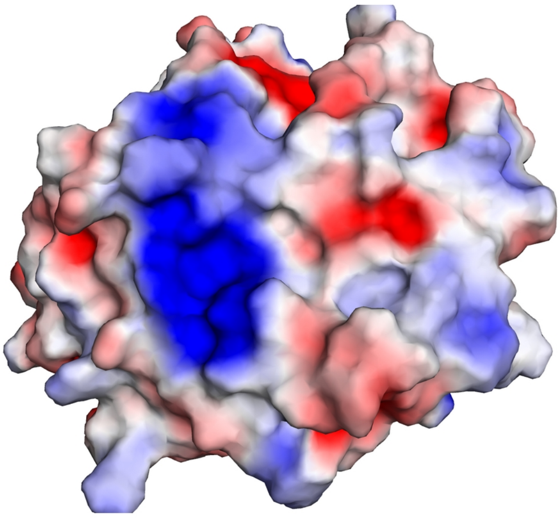

HLA-A1101-BCG_ASGAKTGAK

PAIR #32

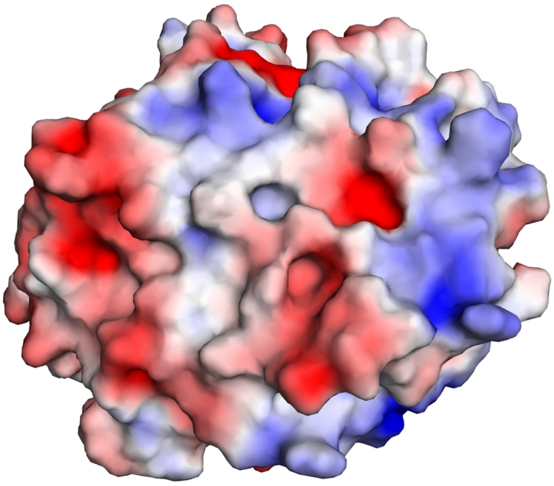

HLA-A2402-SARS_VYFLQSINF

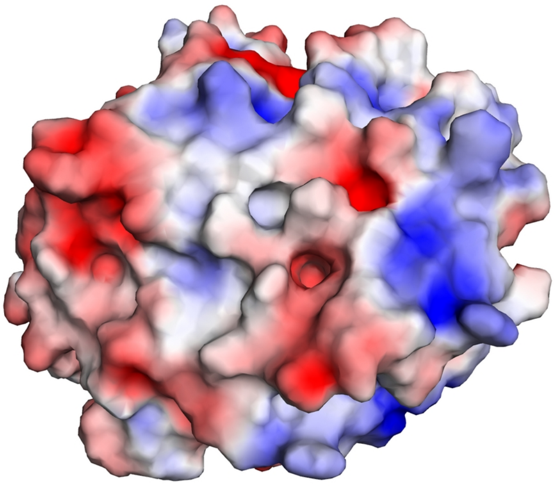

HLA-A2402-BCG_VYSVLLALL

PAIR #33

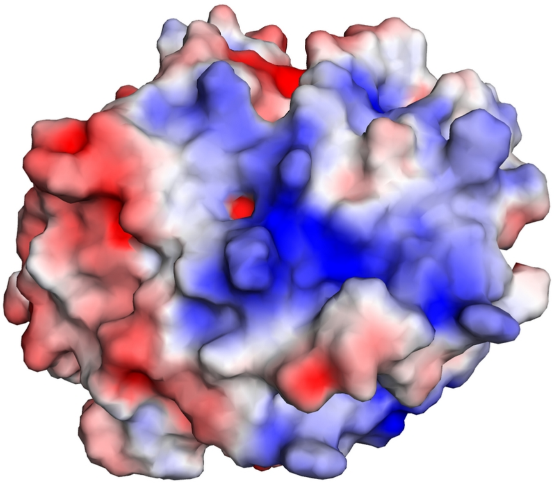

HLA-A2402-SARS_YFVVKRHTF

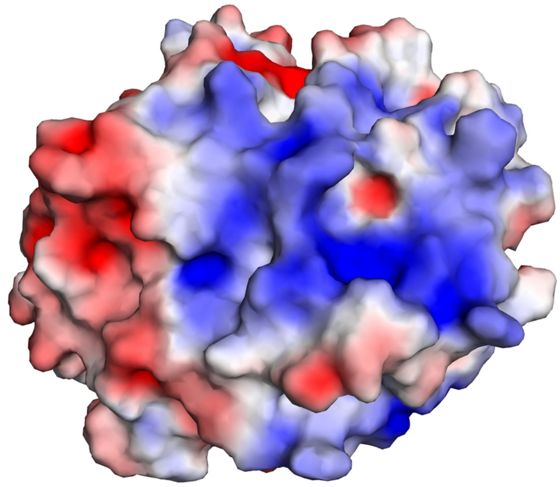

HLA-A2402-BCG_VFPGRKGGF

PAIR #34

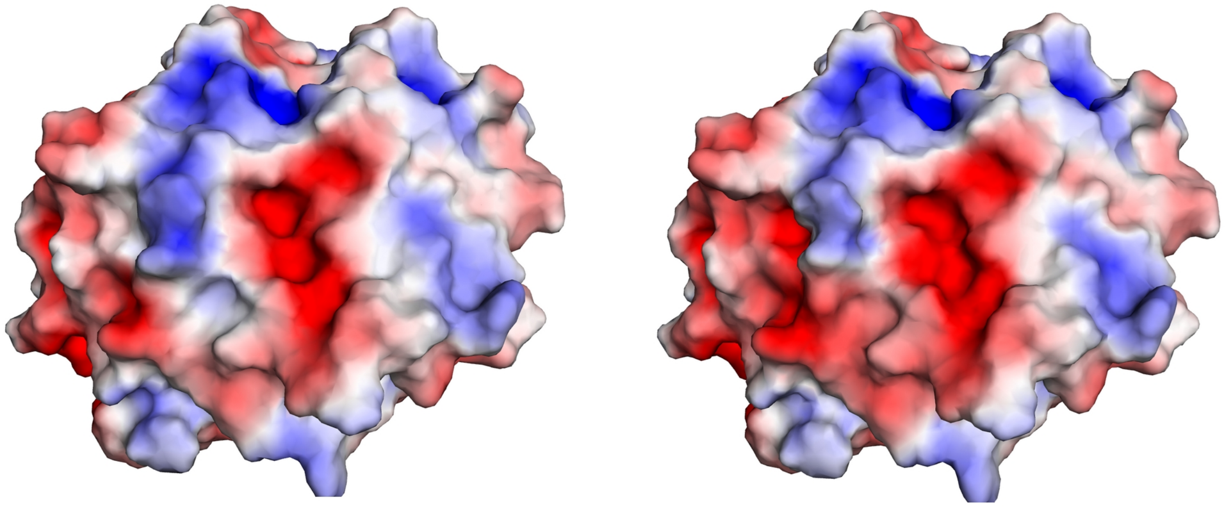

HLA-B0702-SARS_FPRGQGVPI HLA-B0702-BCG_DPRGNPVPL

PAIR #35

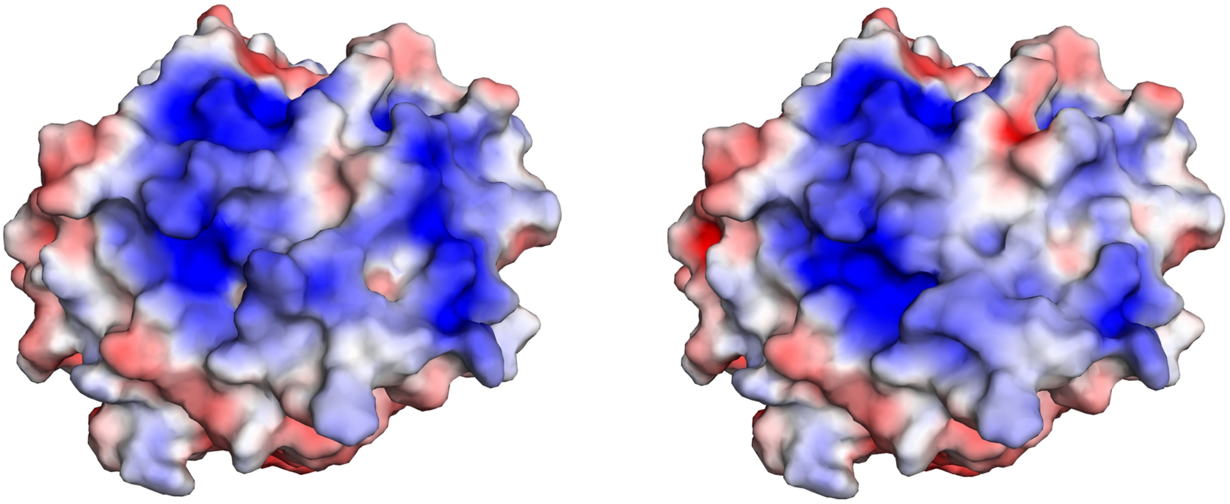

HLA-B3501-SARS_FAYANRNRF HLA-B3501-BCG_MASAARLAA

PAIR #36

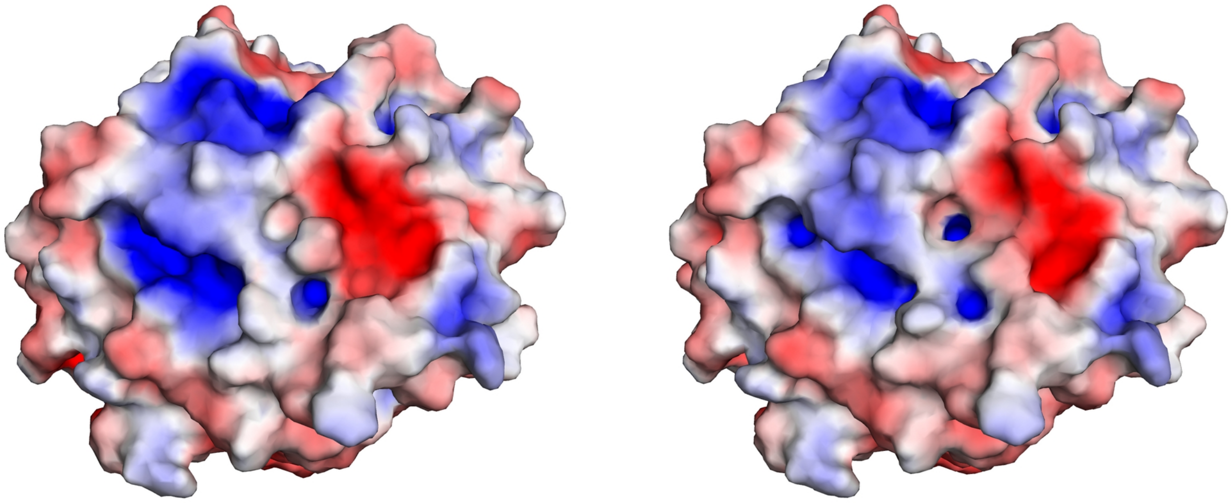

HLA-B3501-SARS_SANNCTFEY HLA-B3501-BCG_QPALFTVEY

PAIR #37

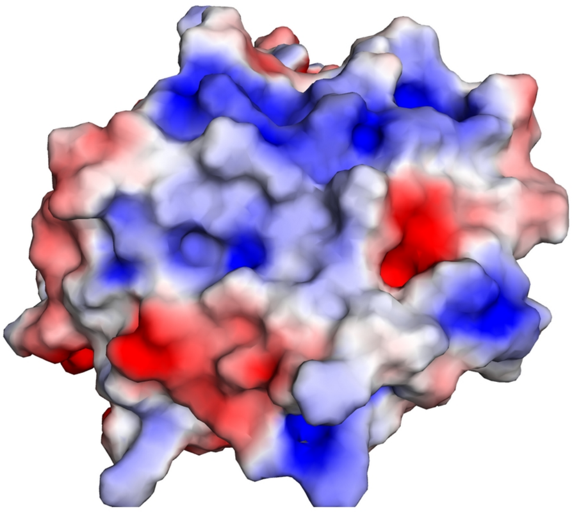

HLA-B4001-SARS_NELSRVLGL

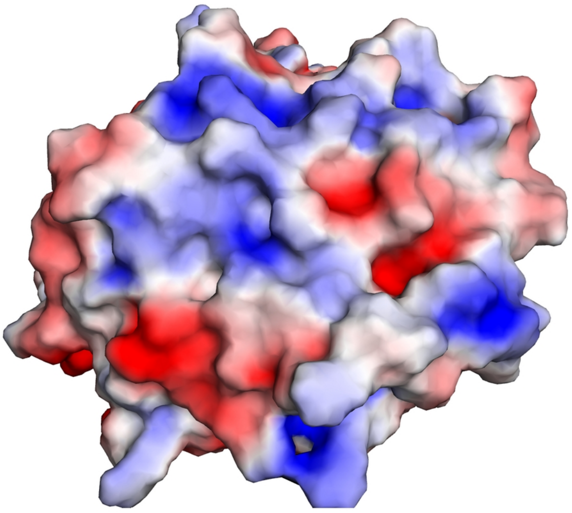

HLA-B4001-BCG_VEGQTNHML

PAIR #38

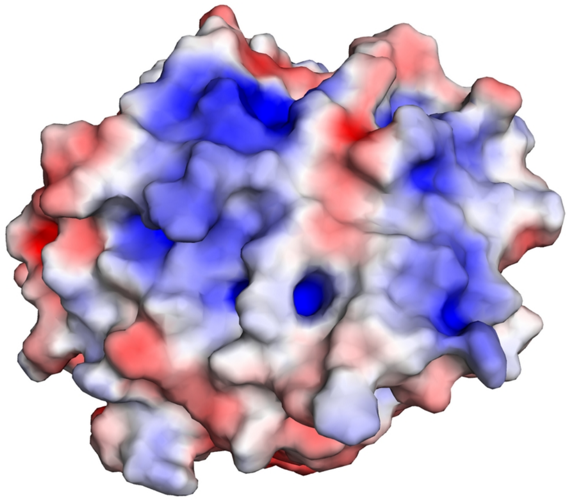

HLA-B3501-SARS_TSNQVAVLY

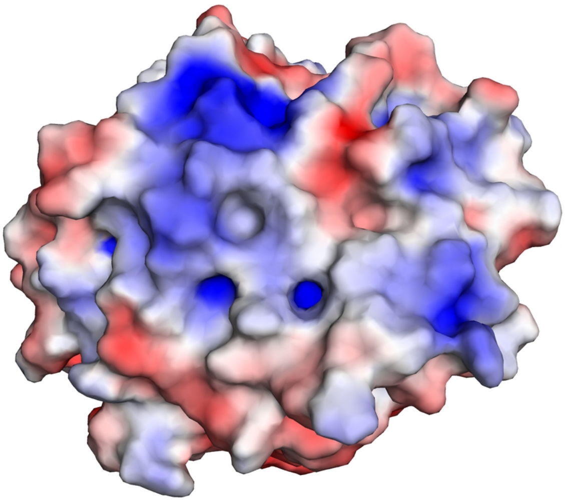

HLA-B3501-BCG_IAAMLLVIY

PAIR #39

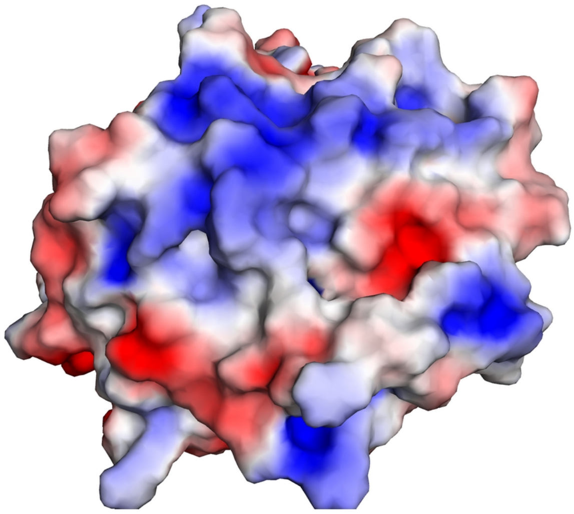

HLA-B4001-SARS_AEIRASANL

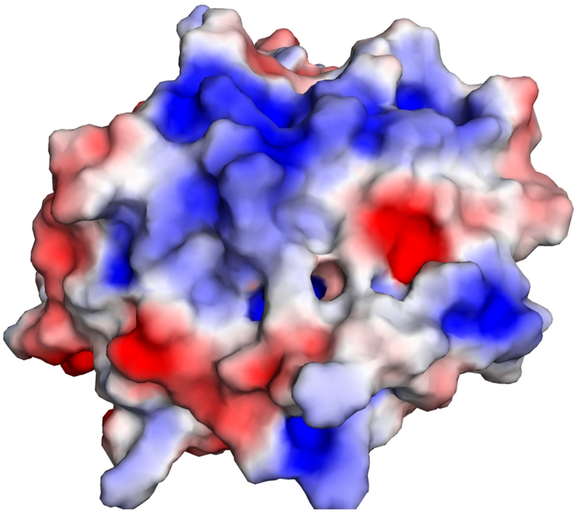

HLA-B4001-BCG_AEPRATGHI

PAIR #40

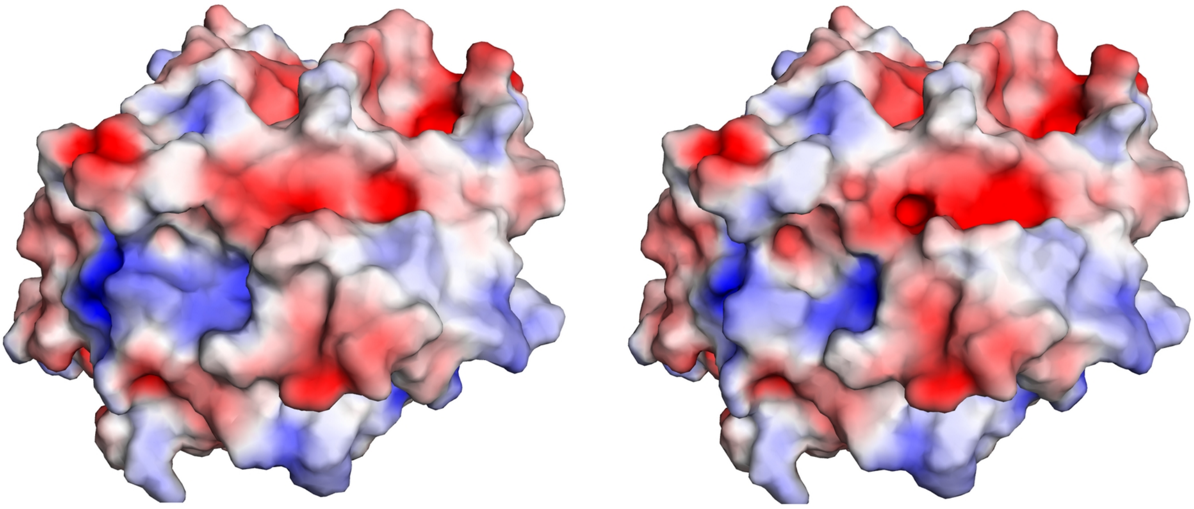

HLA-A0101-SARS_LTDEMIAQY

HLA-A0101-BCG_MTNDNLEYY
